# Targeting *ESR1* mutation–induced transcriptional addiction in breast cancer with BET inhibition

**DOI:** 10.1172/jci.insight.151851

**Published:** 2022-09-08

**Authors:** Sm N. Udden, Qian Wang, Sunil Kumar, Venkat S. Malladi, Shwu-Yuan Wu, Shuguang Wei, Bruce A. Posner, Sophie Geboers, Noelle S. Williams, Yulun Liu, Jayesh K. Sharma, Ram S. Mani, Srinivas Malladi, Karla Parra, Mia Hofstad, Ganesh V. Raj, Jose M. Larios, Reshma Jagsi, Max S. Wicha, Ben Ho Park, Gaorav P. Gupta, Arul M. Chinnaiyan, Cheng-Ming Chiang, Prasanna G. Alluri

**Affiliations:** 1 Department of Radiation Oncology, University of Texas (UT) Southwestern Medical Center, Dallas, Texas, USA.; 2 Department of Radiation Oncology and; 3 Lineberger Comprehensive Cancer Center, University of North Carolina, Chapel Hill, North Carolina, USA.; 4 Department of Bioinformatics,; 5 Department of Biochemistry,; 6 Harold C. Simmons Comprehensive Cancer Center,; 7 Department of Population and Data Sciences,; 8 Department of Pathology, and; 9 Department of Urology, UT Southwestern Medical Center, Dallas, Texas, USA.; 10 Ascension Providence Hospital, Southfield, Michigan, USA.; 11 Department of Radiation Oncology, University of Michigan, Ann Arbor, Michigan, USA.; 12 Department of Internal Medicine, Division of Hematology/Oncology, University of Michigan, Ann Arbor, Michigan, USA.; 13 Vanderbilt-Ingram Cancer Center, Vanderbilt University Medical Center, Nashville, Tennessee, USA.; 14 Michigan Center for Translational Pathology, Department of Pathology, University of Michigan, Ann Arbor, Michigan, USA.; 15 Department of Pharmacology, UT Southwestern Medical Center, Dallas, Texas, USA.

**Keywords:** Oncology, Breast cancer, Epigenetics

## Abstract

Acquired mutations in the ligand-binding domain (LBD) of the gene encoding estrogen receptor α (*ESR1*) are common mechanisms of endocrine therapy resistance in patients with metastatic ER^+^ breast cancer. The *ESR1* Y537S mutation, in particular, is associated with development of resistance to most endocrine therapies used to treat breast cancer. Employing a high-throughput screen of nearly 1,200 Federal Drug Administration–approved (FDA-approved) drugs, we show that OTX015, a bromodomain and extraterminal domain (BET) inhibitor, is one of the top suppressors of *ESR1* mutant cell growth. OTX015 was more efficacious than fulvestrant, a selective ER degrader, in inhibiting *ESR1* mutant xenograft growth. When combined with abemaciclib, a CDK4/6 inhibitor, OTX015 induced more potent tumor regression than current standard-of-care treatment of abemaciclib + fulvestrant. OTX015 has preferential activity against Y537S mutant breast cancer cells and blocks their clonal selection in competition studies with WT cells. Thus, BET inhibition has the potential to both prevent and overcome *ESR1* mutant–induced endocrine therapy resistance in breast cancer.

## Introduction

Approximately 75% of all breast cancers express estrogen receptor α (ERα) and endocrine therapies, which block ERα signaling, are the mainstay of systemic treatment for patients with such cancers (1). Endocrine therapies significantly reduce the incidence, recurrence, and mortality in patients with ER^+^ breast cancers (2). However, nearly all patients with metastatic disease ultimately become refractory to all endocrine therapies (3). Thus, endocrine therapy resistance is the primary obstacle to effective treatment of patients with metastatic ER^+^ breast cancer.

Acquired mutations in the ligand-binding domain (LBD) of the gene encoding ERα (*ESR1*) are some of the most common mechanisms of endocrine therapy resistance in patients with metastatic ER^+^ breast cancer (4–7). *ESR1* mutations are rare in treatment-naive primary tumors and arise under selective pressure of antiestrogen therapy (4, 5). Nearly a third of all patients with metastatic ER^+^ breast cancer harbor *ESR1* mutations (8). Y537S and D538G are the 2 most commonly mutated residues in the LBD of ERα. Collectively, these 2 mutations account for over 70% of all *ESR1* mutations found in breast cancer patients (9). *ESR1* LBD mutations confer estrogen-independent growth and variable resistance to many endocrine therapies (10). The Y537S mutation, in particular, is associated with a higher degree of resistance to most endocrine therapies, among all *ESR1* LBD mutations (8, 10–12). The Y537S mutation drives super-enhancer–mediated transcriptional rewiring of ER^+^ breast cancer cells and is associated with worse clinical outcomes relative to other LBD mutations (8, 11). Therapeutic strategies that preferentially target *ESR1* mutations, particularly Y537S, remain an unmet clinical need. In this study, we explore the therapeutic potential of bromodomain and extraterminal domain (BET) inhibition to target *ESR1* mutation–induced “transcriptional addiction” (11) in ER^+^ breast cancer.

## Results

### ESR1 mutations confer estrogen-independent growth in vivo.

**Figure 1:**
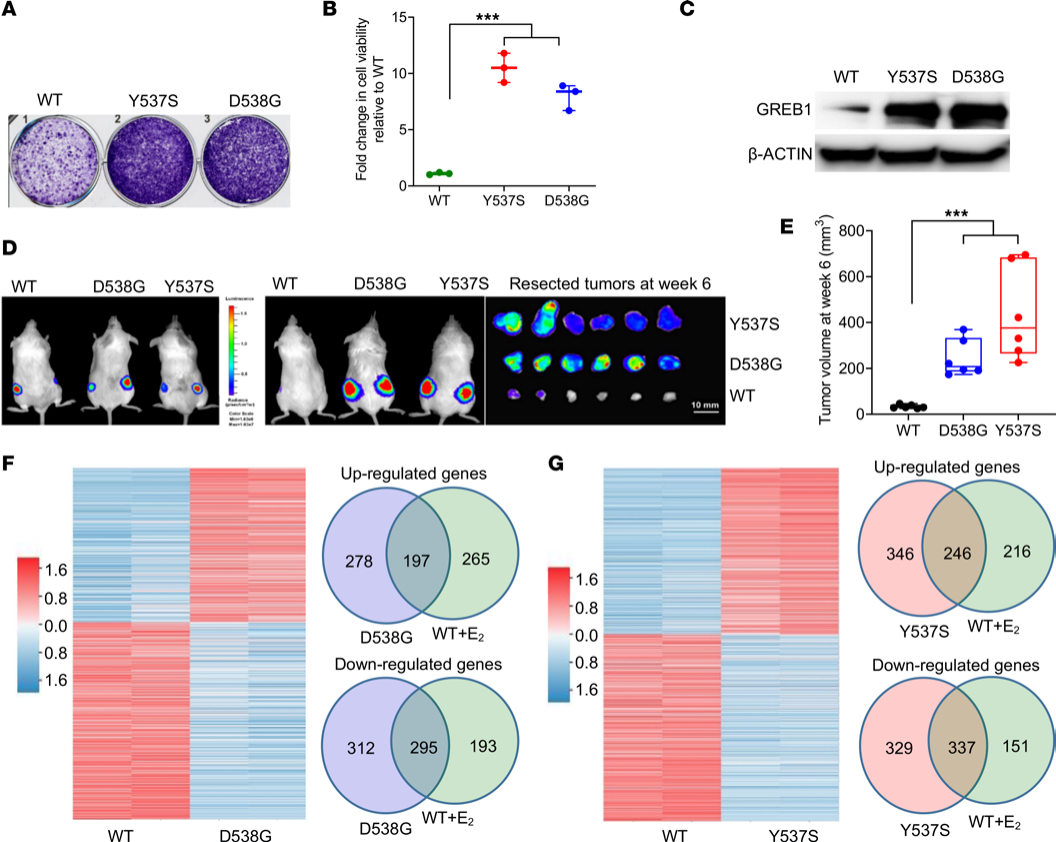
*ESR1* mutations confer estrogen-independent growth in vivo. (**A** and **B**) MCF-7 cells harboring WT or mutant (Y537S or D538G) *ESR1* were hormone deprived for 3 days and plated at a density of 10,000 cells per well in a 6-well plate in triplicate. Cells were allowed to grow for 6 days, and cell viability was quantified by crystal violet staining. (**C**) MCF-7 cells harboring WT or *ESR1* Y537S or D538G mutations were hormone deprived for 3 days, and lysates were immunoblotted as indicated. (**D** and **E**) MCF-7 cells harboring WT, D538G, or Y537S mutations and stably transfected with a luciferase reporter were injected s.c. (5 million cells/injection) in the flanks of ovariectomized, NOD-SCID mice (*n* = 6 tumors) and allowed to grow without exogenous β-estradiol supplementation. Tumors were resected at the end of week 6 (**D**), and tumor volumes were quantified (**E**). Statistical significance was evaluated using ANOVA with Dunnett’s test to adjust for multiple comparisons. ***P ≤ 0.0005. (**F** and **G**) Heatmap showing differentially regulated genes in MCF-7 cells harboring D538G (**F**) or Y537S mutation (**G**) relative to cells harboring WT *ESR1* (FC > 2 and FDR < 0.05) in hormone-depleted conditions. Also shown are Venn diagrams depicting overlap between upregulated/downregulated genes in β-estradiol–stimulated WT cells and hormone-depleted mutant cells as indicated.

To recapitulate the phenotypic characteristics of tumors from ERα^+^ breast cancer patients harboring *ESR1* mutations in a preclinical model, we employed ER^+^ MCF-7 cells that were genome edited using adeno-associated virus (AAV) technology to knock in the 2 most common *ESR1* mutations, Y537S and D538G (13). The mutant allele frequency was 50% for both the mutants, suggesting heterozygous targeting (13). When grown in estrogen-depleted conditions, the mutant cells exhibited high expression levels of GREB1 protein (a marker of activation of ER signaling) and rapid growth. Under the same conditions, control cells harboring the WT *ESR1* showed no detectable expression of GREB1 protein and exhibited growth retardation (Figure 1, A–C). To recapitulate the estrogen-independent growth characteristics of *ESR1* mutant tumors in vivo, we stably transfected a luciferase reporter into MCF-7 cells harboring the WT or mutant (Y537S and D538G) *ESR1* and injected them s.c. into 6- to 8-week-old ovariectomized, female nonobese diabetic severe combined immunodeficient (NOD-SCID) mice without exogenous β-estradiol supplementation. MCF-7 cells harboring *ESR1* mutations supported robust tumor growth, while the corresponding cells harboring WT *ESR1* failed to generate viable tumors (Figure 1, D and E). Exogenous estrogen supplementation rescued the parental MCF-7 cells, and this process supported robust xenograft growth in these conditions (Supplemental Figure 1A; supplemental material available online with this article; https://doi.org/10.1172/jci.insight.151851DS1). These findings were replicated in xenografts derived from T-47D cells harboring WT or *ESR1* mutantations (Y537S and D538G) (Supplemental Figure 1B). Thus, xenografts harboring *ESR1* mutations exhibited substantial growth advantage over those harboring WT *ESR1* in ovariectomized mice. Based on these findings, we conclude that our preclinical model faithfully recapitulates the phenotypic characteristics of tumors from breast cancer patients harboring *ESR1* mutations and represents an excellent model system for therapeutic studies.

To study *ESR1* mutation–induced global transcriptional changes, we carried out RNA-Seq of MCF-7 cells harboring the Y537S or D538G mutation and the corresponding control cells harboring the WT *ESR1* in both hormone-depleted and estradiol-stimulated conditions; these experiments were conducted with biological replicates. In hormone-depleted conditions, the mutant cells exhibited the expression signature of WT cells stimulated with β-estradiol, suggesting constitutive activation of ER signaling (Supplemental Figure 2A). Consistent with prior reports, in the absence of ligand, *ESR1* mutants also activated unique transcriptional programs distinct from WT cells stimulated with β-estradiol (11, 13). Overall, 592 genes were stimulated and 666 genes were repressed over 2-fold in the Y537S mutant cells (relative to WT cells without β-estradiol stimulation), whereas 475 genes were stimulated and 607 genes were repressed over 2-fold in the D538G mutant cells (FDR < 0.05) (Figure 1, F and G, and Supplemental Tables 1 and 2). Approximately 60% of stimulated genes and 50% of repressed genes in the mutant cells overlapped with the corresponding stimulated/repressed genes in the WT cells treated with β-estradiol (Figure 1, F and G). There was greater than 50% overlap between the 2 mutant cells for both stimulated and repressed genes (Supplemental Figure 2B).

### Pharmacological BET inhibition disrupts ESR1 mutant–driven transcriptional programs.

**Figure 2:**
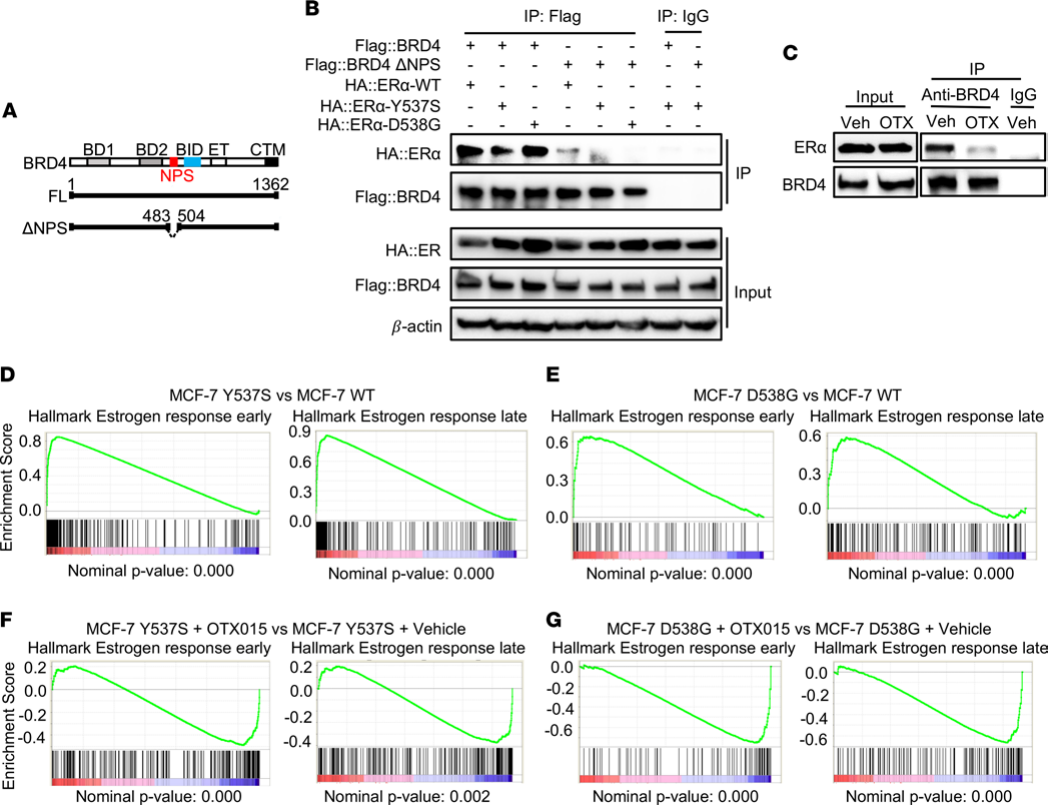
Pharmacological BET inhibition disrupts *ESR1* mutant–driven transcriptional programs. (**A**) Schematic depiction of domain architecture of full length and a deletion construct of BRD4 lacking NPS. BD1, bromodomain I; BD2, bromodomain II; BID, basic residue-enriched interaction domain; ET, extraterminal domain; NPS, N-terminal cluster of phosphorylation sites; CTM, C-terminal motif. (**B**) FLAG-tagged BRD4 (full-length or deletion construct lacking NPS) and HA-tagged WT, Y537S, or D538G ERα were ectopically expressed in MCF-7 cells, and nuclear extracts were subjected to immunoprecipitation using α-FLAG antibody and analyzed for the presence of ERα. (**C**) Nuclear extracts from MCF-7 Y537S cells were subjected to immunoprecipitation with BRD4 antibody and analyzed for the presence of native ERα following treatment with vehicle or OTX015 (1 μM). (**D** and **E**) GSEA of genes differentially expressed in MCF-7 Y537S (**D**) and D538G cells (**E**) relative to the WT cells showing activation of ERα signaling. (**F** and **G**) GSEA of genes differentially expressed in MCF-7 Y537S (**F**) and D538G cells (**G**) following treatment with OTX015 showing suppression of ERα signaling.

BRD4 is a member of the BET family proteins and mediates super-enhancer–driven addiction of tumor cells to oncogenic drivers in a context-dependent fashion (14, 15). Pharmacological BET inhibition promotes loss of BRD4 at super-enhancers and preferentially disrupts transcription of super-enhancer–associated genes (16). Numerous small-molecule BET inhibitors are currently in early-phase clinical trials for treatment of various solid and hematologic malignancies (17). We have previously identified ERα as a binding partner of BRD4 in an unbiased screen of candidate cellular proteins interacting with BRD4 (18). We have also established an essential function for BRD4 in the regulation of ERα-induced gene expression by mediating elongation-associated phosphorylation of RNA polymerase II (19). Since *ESR1* mutations drive super-enhancer–mediated transcriptional reprogramming in ER^+^ breast cancer cells (11), we hypothesized that the transcriptional activity of *ESR1* mutants is likewise dependent on BRD4. To address this question, we first asked if mutant ERα proteins physically interact with BRD4. Using immunoprecipitation, we demonstrate that BRD4 was able to pull down ectopically expressed WT, Y537S, and D538G ER proteins in vivo (Figure 2, A and B). Casein kinase II–mediated phosphorylation of N-terminal cluster of phosphorylation sites (NPS) is critical for binding of BRD4 to acetylated chromatin and recruitment of sequence-specific transcription factors to gene promoters (Figure 2A) (18). Consistent with this, an NPS deletion construct of BRD4 failed to effectively pull down both the WT and mutant ERα proteins, suggesting that NPS is crucial for BRD4 interaction with WT and mutant ERα proteins (Figure 2B). Furthermore, OTX015, a small-molecule BET inhibitor (20, 21), disrupted the native BRD4-ERα interaction in MCF-7 Y537S cells (Figure 2C). Since these cells express both WT and mutant (Y537S) ERα proteins (13), our data suggest that OTX015 disrupts both of these interactions. In summary, WT and mutant ERα proteins both physically interact with BRD4, and pharmacological BET inhibition disrupts this interaction. Next, we used RNA-Seq analysis to study gene expression changes associated with disrupting mutant (Y537S and D538G) ERα–BRD4 interaction with OTX015. Treatment of MCF-7 cells with OTX015 reversed the transcriptional programs mediated by the Y537S and D538G mutations. Thus, in gene set enrichment analyses (GSEA), genes involved in early and late ERα response and MYC targets, which were the top positively enriched signatures in *ESR1* mutant cell lines, were the top negatively enriched signatures following OTX015 treatment (Figure 2, D–G, and Supplemental Figure 3). These findings suggest that BET inhibition disrupts the transcriptional programs driven by *ESR1* mutations.

### OTX015 is one of the top inhibitors of ESR1 mutant cells relative to drugs in the Prestwick chemical library.

**Figure 3:**
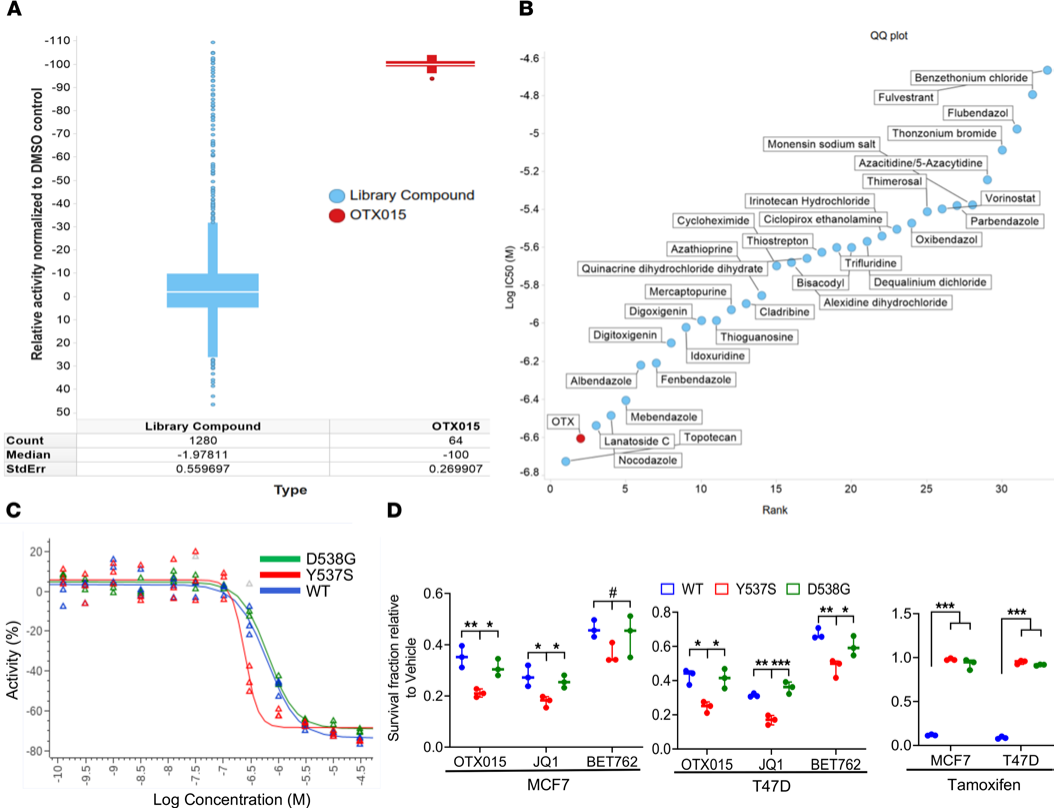
OTX015 is one of the top inhibitors of *ESR1* mutant cells relative to drugs in the Prestwick chemical library. (**A**) A plot of relative activity of individual compounds in the Prestwick chemical library relative to OTX015 performed in MCF-7 Y537S cells. The screen was carried out at a drug concentration of 3 μM in a 384-well format. Cytotoxicity was assayed by luminescence using Cell Titer Glo. (**B**) A plot of log IC_50_ values of the top hits identified in the primary screen. Dose-response relationships for the top hits from the primary screen were established against the WT and the 2 mutant cells in a multi-dose format with 3 replicates per compound per dose per cell line. (**C**) Dose-response relationship curves for OTX015 against MCF-7 cells harboring *ESR1* WT, Y537S, or D538G mutations. (**D**) MCF-7 or T-47D cells harboring WT or mutant (Y537S or D538G) *ESR1* were plated at a density of 10,000 cells per well in a 6-well plate in triplicate. Cells were treated with the indicated drug at 1 μM concentration and allowed to grow for 6 days. Cell viability was quantified by crystal violet staining, and data were plotted as fraction survival relative to vehicle treatment. Statistical significance was evaluated using ANOVA with Dunnett’s test to adjust for multiple comparisons. ***P ≤ 0.0005; **P ≤ 0.005; *P ≤ 0.05 by ANOVA with Dunnett’s test and ^#^*P* ≤ 0.05 by unpaired *t* test.

We next sought to benchmark the growth inhibitory effects of OTX015 in *ESR1* mutant cells against a large library of mostly FDA-approved drugs. For this purpose, we employed the Prestwick chemical library (https://www.prestwickchemical.com/), a collection of 1,280 compounds, approximately 90% of which are FDA-approved drugs, in a primary screen against MCF-7 Y537S and D538G cell lines. Many cytotoxic and noncytotoxic drugs clinically used to treat breast cancer, such as docetaxel, cyclophosphamide, tamoxifen, anastrozole, and fulvestrant, are represented in this drug library. The screening window coefficient (*Z*’ factor) was used to optimize assay conditions (22). Cells were plated in 384-well format, and after incubating the assay plates overnight, 1 drug per well was added to each plate to a final compound concentration of 3 μM. After an incubation period of 96 hours, Cell Titer Glo reagent (Promega) was added to each well, and luminescence was measured. *Z* scores were calculated from the corrected normalized activity for each compound (23). The assays for all cell lines displayed *Z*’ values greater than 0.6, which is considered robust. A plot of activity of each library compound relative to OTX015 was generated. OTX015 emerged as one of the top hits in this primary screen against both the Y537S and D538G cell lines (Figure 3A and Supplemental Figure 4). The top 30–40 hits from the primary screen were further validated in confirmatory studies in the WT as well as the 2 mutant cells in a multidose format, with 3 replicates per compound, per dose, per cell line. OTX015 emerged as one of the top 2 hits against the Y537S mutant cells and among the top 10 hits against the D538G mutant cells (Figure 3B and Supplemental Figure 4B). To determine if OTX015 has preferential activity against the *ESR1* mutant cells, we determined IC_50_ of the drug in MCF-7 cells harboring the WT *ESR1* or Y537S/D538G mutations. While OTX015 exhibited similar activity against both the WT and D538G cells, it showed ~3-fold higher selectivity against the Y537S cells relative to both the WT and D538G cells (Figure 3C). To test the generalizability of our findings, we evaluated the growth inhibitory effects of 3 small-molecule BET inhibitors — OTX015, JQ1, and I-BET762 — against both MCF-7 and T-47D cells harboring the WT or mutant (Y537S or D538G) *ESR1*. All BET inhibitors exerted growth inhibitory effects against MCF-7 and T-47D cells harboring both WT *ESR1* and Y537S/D538G mutations. OTX015 and JQ1 exhibited preferential activity against both MCF-7 and T-47D cells harboring *ESR1* Y537S mutation relative to those harboring WT *ESR1* or D538G mutation (Figure 3D). I-BET762 was less potent than both OTX015 and JQ1 but retained preferential activity against Y537S mutant T-47D cells and a trend toward higher selectivity against Y537S mutant MCF-7 cells relative to the corresponding cells harboring the WT *ESR1* or D538G mutation (Figure 3D). In contrast, tamoxifen, a selective ERα modulator, was completely inactive against both MCF-7 and T-47D cells harboring Y537S and D538G mutations, although it inhibited the growth of corresponding cells harboring the WT *ESR1* (Figure 3D).

### OTX015 inhibits tumor growth in cell line- and patient-derived xenografts harboring ESR1 Y537S mutation.

Since Y537S mutation status is associated with a higher degree of resistance to most endocrine therapies relative to other *ESR1* LBD mutations (8, 10–12), we sought to evaluate the in vivo therapeutic efficacy of OTX015 in xenograft models harboring *ESR1* Y537S mutation. As a first step, we performed pharmacokinetic (PK) studies to determine a dosing regimen for OTX015 in mice that affords drug exposure comparable with human clinical studies (20, 21). A phase Ib study of OTX015 suggested 80 mg once daily with continuous dosing as the recommended dose and schedule for future human efficacy studies (20). Evaluation of human PK at this dose reveal a C_max_ of 1,529 μg/L and an AUC of 11,360 μg × hr/L. An earlier phase I study showed similar results, but they also reported trough values of 165 nM at the same dose (21). In an effort to mimic human exposures in our mouse model, we first evaluated the PK of OTX015 in mice administered orally at 50 mg/kg. Although our sampling was limited, our data at this dose reveal a similar C_max_ to the human data but lower AUC (Supplemental Figure 5). In order to more closely mimic total exposure and keep trough values closer to human values, we elected to dose mice once daily with 100 mg/kg. Prior preclinical studies employing OTX015 have also used this dosing regimen (24).

**Figure 4:**
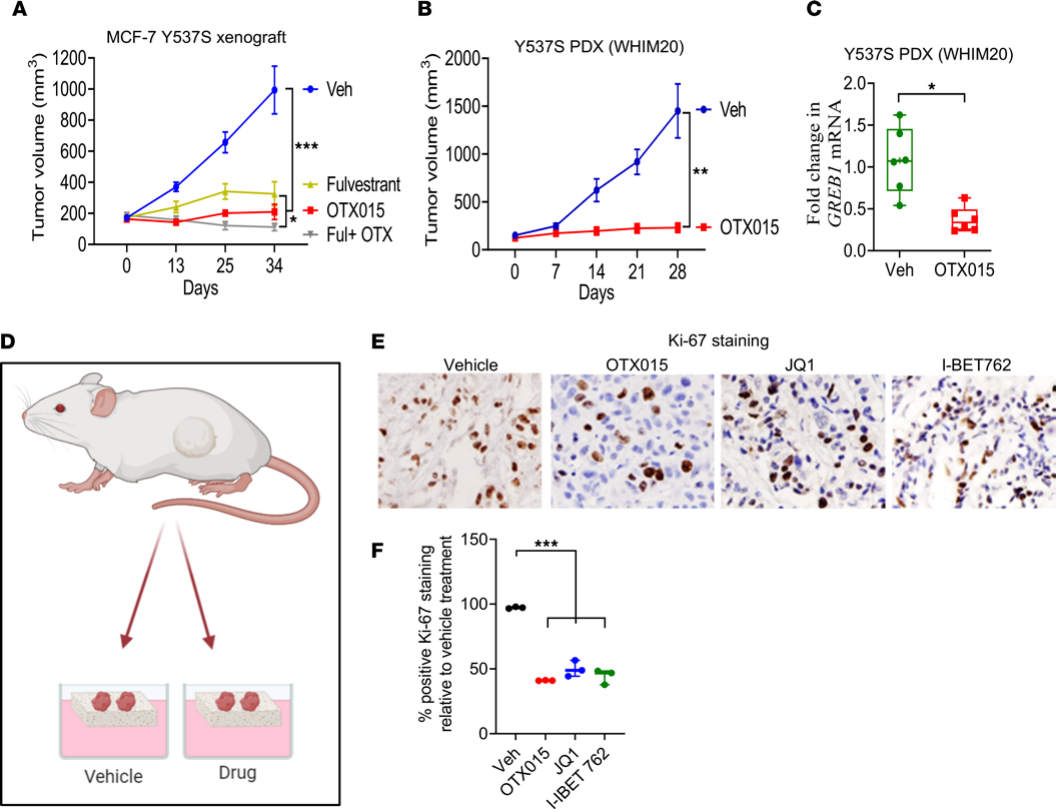
Pharmacological BET inhibition blocks growth of tumor xenografts harboring *ESR1* Y537S mutation. (**A**) MCF-7 Y537S cells were implanted s.c. into ovariectomized athymic, nude mice without exogenous β-estradiol supplementation. When tumors reached 150–200 mm^3^, mice were randomized (*n* = 8 tumors/arm) and received vehicle, OTX015 (100 mg daily by oral gavage, 6 days/week), fulvestrant (1 mg s.c. injection weekly), or OTX015 + fulvestrant as indicated. (**B**) WHIM20 cells harboring Y537S mutation were implanted s.c. into ovariectomized, NOD-SCID mice without exogenous β-estradiol supplementation. When tumors reached 150–200 mm^3^, mice were randomized (*n* = 6 tumors/arm) and received vehicle or OTX015 (100 mg daily by oral gavage, 6 days/week). The results were plotted as average tumor volume measured for each group ± SEM. (**C**) Reverse transcription-PCR for *GREB1* in WHIM20 xenografts (*n* = 6) from vehicle and OTX015-treated mice. (**D**) Schematic representation of patient-derived xenograft explant assay. (**E** and **F**) WHIM20 explants (*n* = 3) were treated with vehicle, OTX015, JQ1, or I-BET762 at a 10 μM concentration for 48 hours, and effect on proliferation was measured by Ki-67 IHC staining. The quantification of the images was performed by the image analysis software ImageJ (NIH), and the results are shown as the ratio between the Ki-67^+^ area and the total area of the image. *P* values were determined using an unpaired, 2-tailed *t* test for pairwise comparisons. To correct for multiple comparisons, ANOVA with Dunnett’s test was used to compare various experimental arms with the vehicle arm. Nonparametric Wilcoxon rank-sum test with Bonferroni correction was used for comparing treatment groups.*P ≤ 0.05; **P ≤ 0.005; ***P ≤ 0.0005.

To evaluate the in vivo efficacy of OTX015, we injected MCF-7 Y537S cells s.c. into the flank of 4- to 6-week-old ovariectomized, athymic nude mice. Mice did not receive exogenous β-estradiol supplementation. When tumors reached a volume of 150–200 mm^3^, mice were randomized to vehicle, OTX015 (100 mg/kg daily as oral gavage, 6 days/week), fulvestrant (1 mg as s.c. injection weekly), or OTX015 + fulvestrant. OTX015 inhibited tumor growth and demonstrated higher efficacy than fulvestrant as a single agent. Combination of OTX015 with fulvestrant did not significantly improve tumor control (Figure 4A). The efficacy of OTX015 as a single agent was further validated in a previously characterized patient-derived xenograft (PDX) harboring *ESR1* Y537S mutation (WHIM20) (25). NOD-SCID mice bearing this xenograft supported estrogen-independent tumor growth, and OTX015 exhibited potent activity in inhibiting tumor growth as a single agent (Figure 4B). OTX015 treatment suppressed mRNA expression levels of *GREB1*, an ER responsive gene, in WHIM20 xenografts, suggesting effective target engagement of the drug in vivo (Figure 4C). We have previously reported optimization of patient-derived explant (PDE) models of hormone-driven cancers to evaluate drug responses in an ex vivo environment, which retains the native tissue architecture and microenvironment of human tumors (26, 27). We adopted this model to evaluate the efficacy of 3 BET inhibitors against explants derived from WHIM20 (Figure 4D). All BET inhibitors evaluated, which included OTX015, JQ1, and I-BET762, exerted greater than 50% inhibition in explant proliferation, as measured by the percentage of Ki-67^+^ cells relative to vehicle treatment (Figure 4, E and F). Thus, our findings show that BET inhibitors, as a class, inhibit *ESR1* Y537S–driven breast cancer growth in in vitro, ex vivo, and in vivo models.

### OTX015 acts synergistically with abemaciclib in inhibiting the growth of MCF-7 cells and xenografts harboring a ESR1 Y537S mutation.

**Figure 5:**
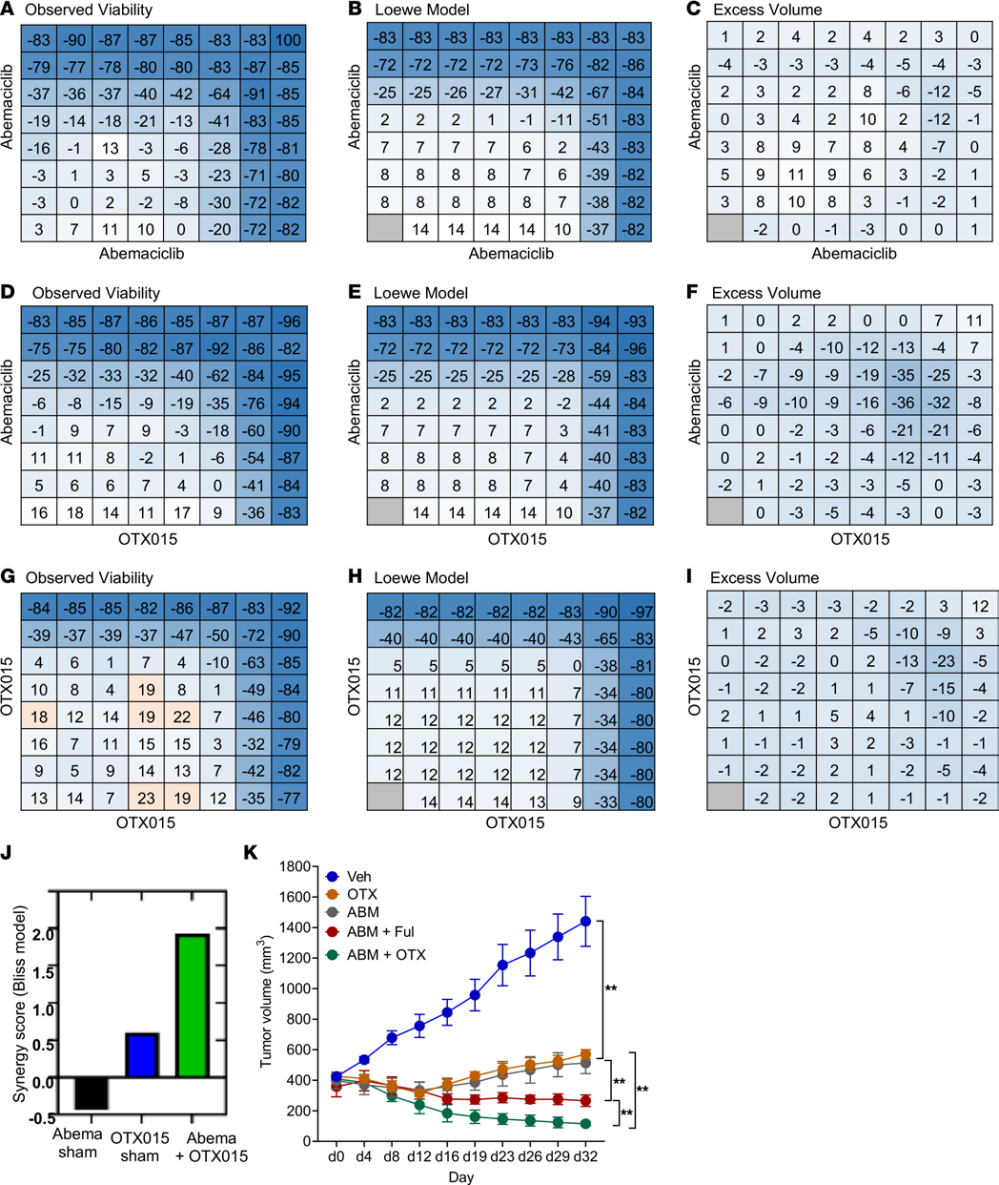
BET inhibition synergizes with abemaciclib in inhibiting the growth of MCF-7 Y537S cells and xenografts. (**A**, **D**, and **G**) Combination matrices were prepared in 384-well microtitier plates, and Y537S MCF-7 cell viability was tested for the following conditions: abemaciclib versus abemaciclib (sham) (**A**), abemaciclib versus OTX15 (**D**), and OTX015 versus OTX015 (sham) (**G**). Concentrations of each drug increase from left to right (0.004, 0.011, 0.033, 0.1, 0.3, 0.9, and 2.7 μM) and from bottom to top (0.004, 0.011, 0.033, 0.1, 0.3, 0.9, and 2.7 μM) with zero drug in the lower left corner of each matrix. (**B**, **E**, and **H**) The additive condition was calculated for each experiment using the Loewe model. (**C**, **F**, **I**, and **J**) Excess volume (observed – model) was calculated for each experiment (**C**, **F**, and **I**), and synergy scores were determined (**J**) as previously described (51). (**K**) MCF-7 Y537S cells were implanted s.c. into athymic, nude mice without exogenous β-estradiol supplementation. When tumors reached 300–400 mm^3^, mice were randomized (*n* = 10 tumors/arm) to receive vehicle, OTX015 (100 mg/kg daily by oral gavage, 6 days/week), abemaciclib (50 mg/kg by oral gavage, 6 days/ week) + fulvestrant (1 mg sub-cutaneous injection weekly), or OTX015 + abemaciclib as indicated. The results were plotted as average tumor volume measured for each group ± SEM. To correct for multiple comparisons, ANOVA with Dunnett’s test was used to compare various experimental arms with the vehicle arm. Nonparametric Wilcoxon rank-sum test with Bonferroni correction was used for comparing treatment groups. **P ≤0.005.

Clinically, CDK4/6 inhibitors are the preferred systemic therapy for the treatment of patients with metastatic ER^+^ breast cancer who have become resistant to endocrine therapies; including through acquisition of *ESR1* mutations (28). Previous studies have shown that BET inhibition synergizes with CDK4/6 inhibitors in NUT midline carcinoma and triple-negative breast cancer through aneuploidy-induced cell cycle arrest (29–31). To test if such synergy exists in the context of *ESR1* mutant breast cancer, we tested OTX015 in combination with abemaciclib, an approved CDK4/6 inhibitor (32), in MCF-7 Y537S cells (Figure 5, A–J). For these studies, we used the Loewe model to evaluate synergy, and we have observed synergy for the abemaciclib/OTX015 combination (synergy score = 1.91) compared with the sham controls for OTX015 (synergy score = 0.6) and abemaciclib (synergy score = –0.435). Synergy was also observed for the abemaciclib/OTX015 combination in MCF-7 cells harboring the WT *ESR1* (synergy score = 2.2) (Supplemental Figure 6) and D538G mutation (synergy score = 1.56) (Supplemental Figure 7). To assess synergy in vivo, we evaluated the efficacy of OTX015 in combination with abemaciclib in ovariectomized, athymic nude mice bearing MCF-7 Y537S xenografts. After tumor implantation, mice were randomized to treatment with vehicle, OTX015 (100 mg/kg daily as oral gavage, 6 days/week), abemaciclib (50 mg/kg daily as oral gavage, 6 days/week), a combination of abemaciclib (50 mg/kg daily as oral gavage, 6 days/week) with fulvestrant (1 mg s.c. injection weekly), or a combination of abemaciclib (50 mg/kg daily as oral gavage, 6 days/week) with OTX015 (100 mg/kg daily as oral gavage, 6 days/week). As expected, OTX015 prevented progression of tumors when used as a single agent (Figure 5K). Combination of abemaciclib with fulvestrant induced 1.3-fold regression in average tumor size. Remarkably, combination of abemaciclib with OTX015 produced 3.5-fold regression in average tumor size and was statistically superior to abemaciclib + fulvestrant (Figure 5K). These findings suggest that OTX015 synergizes with abemaciclib in the treatment of *ESR1* mutant, endocrine therapy–resistant breast cancer, both in vitro and in vivo, and is superior to the current standard of care treatment of combination of CDK4/6 inhibitor with fulvestrant. Thus, our study provides therapeutic rationale for evaluating this combination in future clinical trials.

### Top genes overexpressed in ESR1 mutant cells are also overexpressed in tumors of patients harboring ESR1 mutations and inhibited by OTX015.

**Figure 6:**
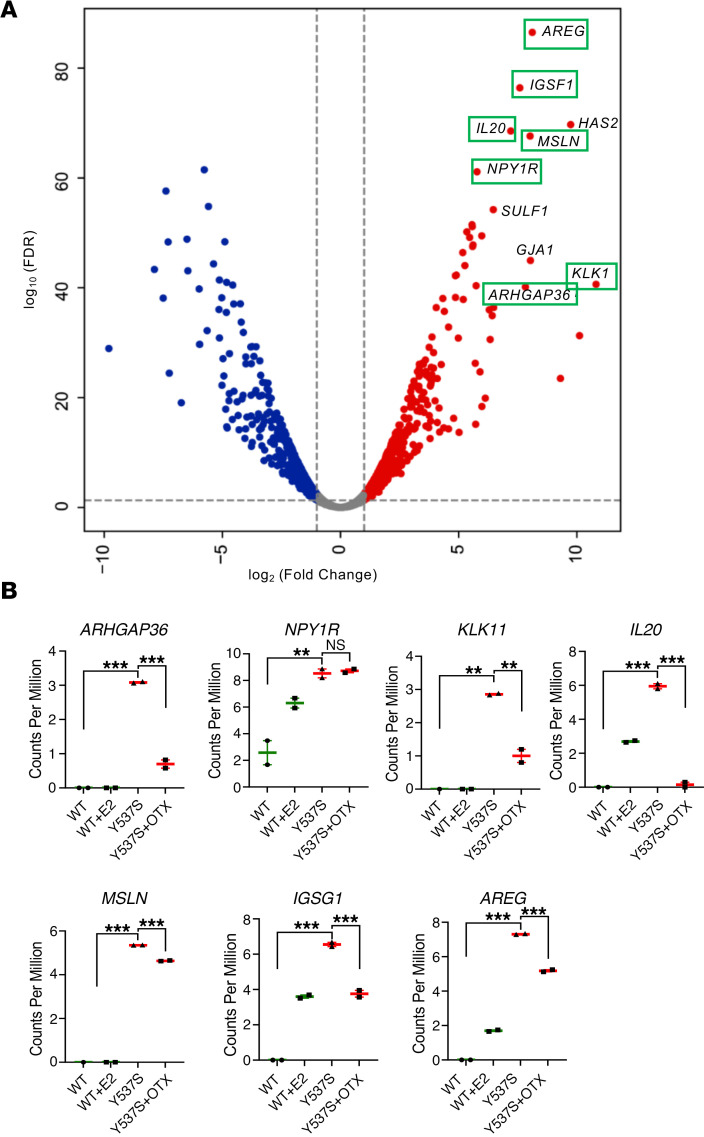
Top genes overexpressed in MCF-7 Y537S cells are also overexpressed in tumors of patients harboring *ESR1* Y537S mutation and inhibited by OTX015. (**A**) A volcano plot depicting genes that are significantly upregulated (red) or downregulated (blue) in MCF-7 Y537S cells relative to WT cells based on log(FC) and FDR thresholds. The top 10 overexpressed genes in the MCF-7 Y537S cells were identified and labeled in the plot. Genes from this top 10 list that were also overexpressed in tumor samples from patients with breast cancerharboring *ESR1* Y537S mutation (11) are highlighted with a green rectangle. (**B**) Expression levels of genes identified above following treatment of MCF-7 Y537S cells with vehicle or OTX015. Statistical significance was evaluated using ANOVA with Dunnett’s test to correct for multiple comparisons. ***P ≤ 0.0005; **P ≤ 0.005.

To determine if the genes overexpressed in *ESR1* Y537S cells are also overexpressed in tumors from breast cancer patients harboring *ESR1* mutations, we integrated the RNA-Seq data from our MCF-7 Y537S cells with previously reported transcriptomic data of tumors from *ESR1* mutant breast cancer patients (11). Jeselsohn et al. employed the Genotype-Tissue Expression (GTex) data set to exclude genes that were upregulated in the corresponding normal tissue of the metastatic site and generated transcriptomic profiles of genes upregulated in human breast tumors harboring *ESR1* LBD mutations relative to WT tumors (11). First, we identified the top 10 genes overexpressed in MCF-7 Y537S cells (relative to control cells harboring WT *ESR1*) based on fold change (FC) and FDR (Figure 6A and Supplemental Table 3). These included a combination of both estrogen-responsive genes and estrogen-independent genes (Figure 6B). Remarkably, 7 of the top 10 genes in Y537S cells were also overexpressed in human breast tumors with the corresponding *ESR1* mutation (Figure 6A) (11). Next, we focused our attention on evaluating the impact of OTX015 treatment on the expression of this subset of 7 genes that were overexpressed in both the MCF-7 Y537S cells and tumors from breast cancer patients harboring the same mutation. In our RNA-Seq analysis, 6 of these 7 genes were inhibited by OTX015 (Figure 6B). These findings reinforce the clinical relevance of the cellular and xenograft models employed in this study and demonstrate that OTX015 inhibits most of the top overexpressing genes in MCF-7 Y537S cells that are also overexpressed in *ESR1* mutant patient tumors (11). Next, we compared the effect of OTX015 treatment on all Y537S-stimulated genes (relative to WT cells) in MCF-7 cells (Group 1) versus genes whose expression was unaltered by the Y537S mutation in the same cells (Group 2). We used a 2 sided *t* test to test the null hypothesis that average FC in expression levels of genes in the 2 groups following OTX015 treatment is not different. OTX015 exhibited remarkable selectivity toward inhibition of genes that are overexpressed in MCF-7 cells harboring the Y537S mutation (*P* = 9.29 × 10^–154^) (Supplemental Table 4). These findings reinforce the view that OTX015 has high selectivity in transcriptionally targeting genes and pathways that are upregulated in the Y537S mutant cells.

### OTX015 has preferential activity against Y537S cells and blocks clonal selection of Y537S mutation in competition studies.

**Figure 7:**
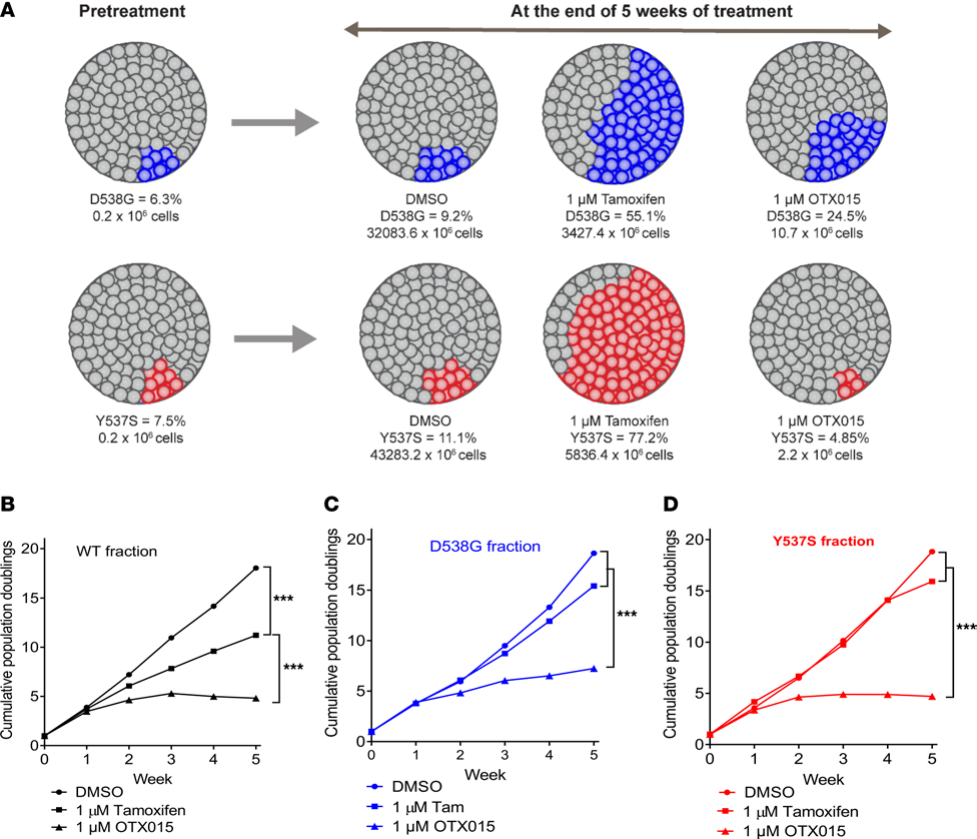
OTX015 suppresses growth of T-47D cells expressing *ESR1* Y537S mutation. (**A**) Schematic representations of competition-based growth analyses performed using mixtures of T-47D cells generated by spike-in of CRISPR-engineered D538G (upper panel, blue) or Y537S (lower panel, red) clones into the nonmutated parental T-47D cell line. Pretreatment mutant allele frequency (MAF) was quantified by duplex digital PCR (dPCR), and total counts of mutant cell clones are also indicated. These cell line mixtures were cultured for 5 weeks in the presence of either DMSO, 1 μM tamoxifen, or 1 μM OTX015. The endpoint allele frequencies for D538G (upper panels, blue) or Y537S (lower panels, red) and the total cell numbers of the mutant clones are indicated for the endpoint of the drug treatment study. (**B**–**D**) Analysis of MAF and total cell counts were used to generate growth curves or cumulative population doublings for *ESR1*-WT (black) (**B**), *ESR1*-D538G (blue) (**C**), and *ESR1* Y537S (red) (**D**) clones during exposure to DMSO, 1 μM tamoxifen, or 1 μM (OTX015). To correct for multiple comparisons, ANOVA with Dunnett’s test was used to compare various experimental arms with the vehicle arm. Nonparametric Wilcoxon rank-sum test with Bonferroni correction was used for comparing treatment groups. ***P ≤ 0.0005. Representative results from 1 of 3 independent experiments are shown.

Previous preclinical and clinical studies have demonstrated that Y537S mutation status is associated with a higher degree of resistance to endocrine therapies used for treating ER^+^ breast cancer, such as aromatase inhibitors, tamoxifen, and fulvestrant, relative to other *ESR1* LBD mutations (4, 5, 8, 10–12, 33). To our knowledge, drugs that preferentially target Y537S mutant cells have not been previously described. To investigate if the higher selectivity of OTX015 against the Y537S cells (relative to WT cells) results in a negative selection pressure against this mutation in competition studies, we mixed 6%–8% of T-47D cells harboring homozygous knock-in of the D538G or Y537S mutation with WT cells and treated them with vehicle, tamoxifen, or OTX015. Tamoxifen treatment resulted in an approximately 9-fold and 7-fold reduction in overall number of D538G/WT and Y537S/WT cells, respectively. However, tamoxifen exerted a positive selection pressure on both mutant cells. Thus, the mutant fraction of D538G and Y537S increased to approximately 55% (from 6.3%) and 77% (from 7.5%), respectively (Figure 7). These findings suggest that tamoxifen treatment accelerates emergence of *ESR1* mutation-induced endocrine therapy resistance by exerting a positive selection pressure on the mutant cells. OTX015, on the other hand, exerted nearly 3,000- and 20,000-fold reduction in D538G/WT and Y537S/WT cells, respectively. OTX015 treatment led to lesser degree of enrichment of D538 mutation compared with tamoxifen (24.5% versus 55.1%). Remarkably, OTX015 treatment completely eliminated enrichment of Y537S mutation (7.5% before treatment versus 4.85% after treatment with OTX015 versus 77.2% after treatment with tamoxifen) (Figure 7). Our data suggest that, while BET inhibition blocks the transcriptional function of both WT and mutant ERα proteins, it represents a preferential vulnerability for the Y537S mutation. These findings are consistent with our data from growth inhibition assays, which show that the IC_50_ of OTX015 is ~3 fold more potent in Y537S cells relative to WT and D538G cells. While the mechanistic underpinnings for higher selectivity of BET inhibitors toward the Y537S mutant breast cancer cells remain to be elucidated, Jeselsohn and coworkers have previously shown that Y537S mutation activates unique transcriptional programs relative to other *ESR1* mutations (and WT cells stimulated with β-estradiol) (11). Thus, mutant ER was recruited to 35,000 DNA binding sites in MCF-7 Y537S cells and to 11,371 sites in D538G mutant cells. Furthermore, ER binding sites gained in Y537S cells are more likely to occur in promoter regions relative to other cells. Finally, over 30% of super-enhancers found in the Y537S cells overlapped with mutant gained DNA-binding sites. Thus, Y537S mutant–driven transcriptional programs may be more reliant on super-enhancers than those driven by WT or D538G mutant ER proteins. These findings are consistent with our data, which show that Y537S mutation activates unique transcriptional programs relative to both D538G mutation and WT ER stimulated with estrogen (Figure 1 and Supplemental Figure 2). Since BET inhibition has higher selectivity for targeting super-enhancer–driven transcriptional programs (14), this may explain the preferential activity of OTX015 toward Y537S cells.

This finding has significant clinical relevance, as the Y537S mutation status is associated with a higher degree of resistance (relative to other *ESR1* LBD mutations) to nearly all endocrine therapies clinically used in the treatment of ERα^+^ breast cancer and as development of drugs that preferentially target the Y537S mutation remains an acute unmet clinical need.

## Discussion

Somatic mutations in the LBD of *ESR1* are one of the most common mechanisms of acquired resistance to endocrine therapies (34). *ESR1* mutations are found in nearly one-third of all patients with metastatic ER^+^ breast cancer and are associated with worse clinical outcomes, including overall survival (4, 5, 8, 33). *ESR1* mutations are also enriched in patients with localized breast cancer receiving neoadjuvant endocrine therapy (35). A recent study has shown that nearly one-third of locoregional recurrent tumors in patients who have completed definitive treatment for ER^+^ breast cancer also harbor *ESR1* mutations (36).

In biochemical studies, the Y537S mutation exhibits the highest degree of transcriptional activity, phosphorylation, and estrogen-independent growth relative to all other LBD mutations (10). In in vivo studies, the Y537S mutation also confers a greater degree of resistance to both tamoxifen and fulvestrant (10, 11). Consistent with these findings, Y537S mutation status is associated with worse overall survival relative to other *ESR1* LBD mutations (8). Furthermore, in large clinical studies such as the PALOMA-3 trial, there was significant enrichment for Y537S mutation at the end of treatment in both fulvestrant + placebo and fulvestrant + palbociclib arms relative to day 1. No such enrichment was found for the remaining 12 *ESR1* mutations evaluated in this study. While more definitive studies are needed, these findings suggest that Y537S mutation status is associated with resistance to both fulvestrant as a single agent and combination of fulvestrant with palbociclib (12). Similarly, in the BOLERO-2 trial, although patients with both the WT and D538G mutation showed a significant improvement in progression-free survival with the addition of everolimus to exemestane, patients harboring the Y537S mutation did not derive any benefit (although the sample size for this subgroup was modest to draw a definitive conclusion) (8). Taken together, retrospective analyses of clinical outcomes from PALOMA-3 and BOLERO-2 trials suggest that the Y537S mutation status may be associated with a higher degree of resistance to both single-agent endocrine therapies and to combination regimens of endocrine therapies with palbociclib and everolimus. However, more definitive studies are needed to establish the role of Y537S mutation in conferring resistance to nonendocrine therapies such as palbociclib and everolimus. Nevertheless, while a number of therapeutics with improved efficacy against *ESR1* mutant breast cancer are being developed, to our knowledge, drugs with preferential activity against the Y537S mutant tumors have not been described.

Many drugs that show promise in preclinical models often fail to afford meaningful clinical benefit in patients. Some key reasons for this high rate of failure include the use of preclinical models of breast cancer that are not clinically relevant, inability to match the right drug to the right patient due to lack of predictive biomarkers, failure to identify rational drug combinations that enhance the clinical utility of the drug, an unfavorable PK profile and an unfavorable toxicity profile. In this study, we employ clinically relevant models of breast cancer that faithfully recapitulate phenotypic characteristics of tumors from patients with treatment-resistant breast cancer. We match these cellular models with a PDX that have the same phenotypic and molecular characteristics to perform mechanistic and therapeutic studies. Using a combination of biochemical and genomic studies, we show that both Y537S and D538G mutant proteins physically interact with BRD4. Treatment of these cells with OTX015, a small molecule BET inhibitor, reverses the transcriptional programs induced by the mutants. By benchmarking its activity against the Prestwick chemical library, we show that OTX015 is one of the top inhibitors of Y537S and D538G mutant breast cancer cells among a large collection of nearly 1200 FDA-approved drugs. By performing careful PK studies, we selected drug exposures that are easily achievable without significant adverse effects in patients (based on human data from 2 phase I clinical trials of OTX015). In cell line and PDX models, OTX015 showed efficacy as a single agent in suppressing tumor growth. Furthermore, OTX015 exhibits synergy when combined with the CDK4/6 inhibitor abemaciclib and induced higher tumor regression than the current standard-of-care regimen of abemaciclib + fulvestrant. Thus, our findings support clinical evaluation of this drug combination in future BET inhibitor trials. By integrating the RNA-Seq data from the MCF-7 Y537S cell lines with transcriptomic data of ER^+^ tumors from breast cancer patients harboring the same *ESR1* mutation (11), we show that many of the top genes overexpressed in the mutant cell line are also overexpressed in patient samples and are targeted for inhibition by OTX015. We also show that transcriptional programs activated by the Y537S mutation confer preferential vulnerability to BET inhibition. Thus, the IC_50_ of OTX015 was ~3-fold more potent for Y537S cells relative to both WT and D538G cells. Consequently, in in vitro evolution experiments, OTX015 treatment completely blocks enrichment of Y537S mutant cells, while tamoxifen treatment results in profound enrichment of these cells. Our findings uncover BET inhibition as a selective vulnerability for *ESR1* Y537S, a mutation associated with a higher degree of resistance to nearly all clinically approved endocrine therapies. Although a number of therapeutics with improved efficacy against *ESR1* mutant breast cancer are being developed, to our knowledge, OTX015 is the only drug with preferential activity against the Y537S mutant breast cancer cells. Thus, our findings support evaluation of Y537S mutation as a predictive biomarker for response in ongoing BET inhibitor trials in ER^+^ breast cancer. Based on their preferential activity against Y537S mutant breast cancer cells, we hypothesize that BET inhibitors may prevent or delay development of Y537S-mediated endocrine therapy resistance and disease recurrence when used in the adjuvant setting for treatment of patients with localized ER^+^ breast cancer. This hypothesis needs to be validated in future clinical studies.

Numerous small-molecule BET inhibitors are currently in early phase clinical trials for treatment of various solid and hematologic malignancies (17). While no efficacy data of BET inhibitors in breast cancer are currently available, a combined phase I/II dose-escalation and expansion study of the BET inhibitor GSK525762 in patients with metastatic ER^+^ breast cancer is currently underway (ClinicalTrials.gov; NCT02964507). OTX015 has shown a favorable safety profile in phase I clinical trials (20, 21). At the recommended 80 mg once-daily continuous dosing schedule for future human efficacy studies, reversible thrombocytopenia (16% of patients) and elevation of alanine transaminase (ALT)/hyperbilirubinemia (5% of patients) were the major dose-limiting toxicities observed in the study. Other treatment-related adverse effects were primarily gastrointestinal in nature (diarrhea, nausea, vomiting, and decreased appetite). Overall, the drug was well tolerated, and the toxicity profile is comparable with other nonendocrine-targeted therapies in breast cancer, such as CDK4/6 inhibitors (37). While these toxicities were reversible and did not impact tolerance in patients, synergistic combinations that allow deescalation of BET inhibitor dose while maintaining or improving efficacy may further minimize toxicity in patients. Interestingly, a combination of OTX015 and abemaciclib (the most potent of the 3 clinically approved CDK4/6 inhibitors) exhibited remarkable efficacy and superior tumor regression compared with the current standard-of-care treatment of abemaciclib + fulvestrant. Our findings provide therapeutic rationale for evaluating this combination in future BET inhibitor clinical trials.

A new-generation BET inhibitor — ABBV-744, targeting BD2 but not BD1 of BRD4 with significantly fewer platelet and gastrointestinal toxicities but with retained efficacy — has recently been described (38). Furthermore, with our recent report describing opposing functions of BRD4 protein isoforms in breast carcinogenesis (39), we have laid the foundation for developing therapeutic approaches for isoform-specific targeting of BRD4. Efforts are underway to define the role of BRD4 protein isoforms in mediating *ESR1* mutant–induced transcriptional dysregulation. We expect that these efforts will lead to development of isoform-specific BRD4 inhibitors with improved efficacy and safety relative to pan-BRD4 inhibitors. Thus, the field of BET inhibitors will continue to evolve; strong mechanism-based translational studies that identify predictive biomarkers for improved response (such as Y537S mutation status) and rational combination treatments (such as CDK4/6 inhibitors) that synergize with BET inhibition and increase response rates will determine the clinical success of this class of drugs for treatment of endocrine therapy–resistant breast cancer.

## Methods

### Cell culture.

T-47D and MCF-7cells were purchased from American Type Culture Collection (ATTC). T-47D cells were cultured in RPMI Medium (Thermo Fisher Scientific) supplemented with 10% FBS from Hyclone. MCF-7 cells were cultured in DMEM (Thermo Fisher Scientific) supplemented with 10% FBS. Cells were grown in a humidified incubator with 5% CO_2_. Cells were assessed for their viability and counted with a Countess II FL Automated Cell Counter (Invitrogen). Monthly mycoplasma screening was performed with PlasmoTest (Invivogen).

### Chemicals/drugs.

Tamoxifen and β-estradiol were purchased from MilliporeSigma. β-Estradiol pellets (0.17 mg, 2-week release) for s.c. implantation were purchased from Innovation Research of America. OTX015, JQ1, I-BET762, fulvestrant, and abemaciclib were purchased from Abmole.

### Generation of ESR1 mutant cell lines.

Generation of genome-edited *ESR1* mutant MCF-7 cells has been previously described (13). *ESR1* mutant T-47D cell lines were generated by CRISPR-Cas9 editing. The Neon Transfection System was used for the delivery of a plasmid containing *ESR1*-targeting sgRNA (5′-GCCCCTCTATGACCTGCTGC-3′; cloned into pGL3-U6 plasmid at BsaI site), Cas9 expression plasmid (Addgene, 44758), and single-stranded oligonucleotide donor templates into T-47D cells following manufacturer guidelines. Successfully targeted cell clones were selected by treatment with 50 nM fulvestrant and genotyped using digital PCR (dPCR) (see below). Competition experiments were conducted by mixing parental T-47D cells with T-47D cells with mutant *ESR1* alleles of interest. Cell mixtures were grown for 5 weeks in the presence of DMSO, tamoxifen (1 μM), or OTX015 (1 μM). A portion of the cell population was harvested each week to measure changes in mutant allele frequency by dPCR.

### Analysis of ESR1 mutations by dPCR.

A duplex dPCR assay was developed to detect WT *ESR1* allele and 2 hotspot *ESR1* mutations, p.D538G or p.Y537S. The duplex assay included a TET conjugated locked nucleic acids–modified (LNA-modified) DNA-oligonucleotide probe that recognized the WT *ESR1* allele (5′-TCTATGACCTG-3′) and FAM conjugated LNA probe targeting either p.D538G (5′-CTATGGCCTGC-3′) or p.Y537S (5′-CCCTCTCTGACCT-3′). The probes were synthesized by IDT (Integrated DNA Technology). Specificity of these probes has been validated previously (40). The 20 μL dPCR assay mixture contained 10 μL of 2× dPCR Supermix for Probes (no dUTP), 0.9 mmol/L of forward (5′-GTCTTCCCACCTACAGTAACAAAGG-3′) and reverse (5′-CTAGTGGGCGCATGTAGGC-3′) primers, 0.25 mmol/L of respective probes, and approximately 100 ng of genomic DNA. dPCR assays were performed on the QX200 platform outfitted with an automated droplet generator (Bio-Rad). After droplet generation, the reactions were subjected to PCR amplification in a thermocycler (Bio-Rad). The PCR cycling parameters were 10 minutes at 95°C followed by 40 cycles of 94°C for 30 seconds, 60°C for 30 seconds, and 72°C for 2 minutes with 2°C/second ramp at all steps. After PCR amplification, the emulsion was transferred to the QX200 instrument (Bio-Rad) to measure the end-point fluorescence signal in each droplet. The dPCR data were analyzed using QuantaSoft software version 1.7.4.0917 (Bio-Rad). Two-dimensional (FAM and HEX intensity) plots were made for each sample, and “gates” were used to define graphical areas with specific fluorescence properties. The number of droplet events specific for WT or mutant *ESR1* alleles was used to calculate the mutation frequency. D538G_ssDNA: 5′-GGCTAGTGGGCGCATGTAGGCGGTGGGCGTCCAGCATCTCAAGCAGCAGGCCATAGAGGGGCACCACGTTCTTGCACTTCATGCTGTACAGATGCTCCATG-3′; Y537S_ssDNA, 5′-TAGTGGGCGCATGTAGGCGGTGGGCGTCCAGCATCTCAAGCAGCAGGTCAGAGAGGGGCACCACGTTCTTGCACTTCATGCTGTACAGATGCTCCATGCCT-3′.

### Hormone depletion and RNA-Seq.

For hormone depletion, cells were cultured in phenol red–free tissue culture medium, IMEM (Thermo Fisher Scientific, A10488-01), supplemented with 5% charcoal-dextran-treated calf serum (Valley Biomedical, catalog BS3050) and 2% penicillin-streptomycin in a T150 flask. Hormone depletion was carried over 3 days by washing the cells with phenol red–free IMEM without serum (5 washings daily, 1 hour apart). At completion of washings each day, cells were recultured in phenol red–free IMEM supplemented with 5% charcoal-dextran-treated calf serum. On day 4, the cells were trypsinized, and 1 × 10^6^ cells were seeded on 10 cm culture dish. After overnight culture, cells were treated with vehicle, 10 pM β-estradiol, or 1μM OTX015 for 24 hours. The cells were then harvested, and total RNA was extracted using RNeasy Mini Kit (QIAGEN) according to the manufacturer’s instructions. RNA quantity and purity was measured by a NanoDrop 2000 spectrophotometer (Thermo Fisher Scientific), and integrity was analyzed by an Agilent Bioanalyzer 2100 (Agilent Technologies). RNA-Seq libraries were prepared using Illumina TruSeq Standard mRNA Sample Preparation Kit (Illumina) according to the manufacturer’s protocol. Libraries were validated on an Agilent Bioanalyzer 2100. RNA-Seq libraries were sequenced on a SE75 (single end 75 bp) NextSeq500 flow cell. The GEO accession number for RNA-Seq data is GSE206185.

### RNA-Seq analysis.

Reads with phred quality scores less than 20 bp and less than 35 bp after trimming were removed from further analysis using trimgalore (v0.4.1). Quality-filtered reads were then aligned to the mouse reference genome GRCh38 (hg38) using the HISAT (v 2.0.1) aligner (41), using default settings and marked duplicates using Sambamba (v0.6.6) (42). Aligned reads were quantified using featurecount (v1.4.6) (43) per gene ID against GENCODE v25 (44). Differential gene expression analysis was done using the R package edgeR (v3.10.5) (45). Cutoff values of absolute FC greater than 2.0 and FDR ≤ 0.05 were then used to select for differentially expressed genes between sample group comparisons. GSEA was assessed on DEGs to the Hallmark (H, v5.1) curated gene set from Molecular Signature Database (https://www.gsea-msigdb.org/gsea/index.jsp). FDR of 0.2 or less was considered statistically significant for GSEA analysis (46). Top-ranking genes in MCF-7 Y537S cells were nominated based on FDR and FC (relative to the expression of same genes in MCF-7 cells harboring WT *ESR1*). Ranking was created using the following formula: –log_10_(FDR) × log_2_(FC) (47).

### Cell growth assays.

For cell growth assays, cells were cultured in phenol red–free IMEM supplemented with 5% charcoal-dextran-treated calf serum (Valley Biomedical, BS3050) and 2% penicillin-streptomycin in a T150 flask. Where indicated, hormone depletion was carried over 3 days as described in the previous section. On day 4, cells were trypsinized, and 1 × 10^4^ cells were seeded in a 6-well tissue culture plate and allowed to grow in an incubator for 6 days. On day 6, cells were washed, fixed with 4% paraformaldehyde, stained with 0.5% crystal violet solution for 20 minutes, washed with water, and air dried. After photographic images of the plates were obtained, the crystal violet was solubilized with 20% acetic acid, and absorbance was measured at 490 nm using a Spark 10M multimode microplate reader (Tecan).

### Immunoprecipitation and immunoblotting.

MCF-7 cells were transfected with FLAG-BRD4 and HA-ERα (WT, Y537S, or D538G). Forty-eight hours after transfection, cells were homogenized in modified RIPA lysis buffer containing 50 mM Tris-HCl (pH 7.5), 1% NP-40, 0.25% sodium deoxycholate, 150 mM NaCl, 1 mM EDTA, complete protease inhibitor cocktail, and phosphatase inhibitor cocktail (Roche). Immunoprecipitation was performed with mouse anti-FLAG antibody (MilliporeSigma, F3165), resolved by SDS-PAGE, and transferred onto a PVDF membrane. The membranes were immunoblotted with antibodies against HA (Cell Signaling Technology, catalog 3724) and anti-FLAG (MilliporeSigma, catalog F3165). Immunoreactive proteins were detected using ECL SuperSignal West Femto substrate reagent (Thermo Fisher Scientific). For endogenous immunoprecipitation and immunoblotting experiments, similar protocol was employed in native MCF-7 Y537S cells, and immunoprecipitation and immunoblotting were performed with antibodies against endogenous ERα (Cell Signaling Technology, catalog 8644) and BRD4 (Cell Signaling Technology, catalog 13440).

### High-throughput drug screen and cytotoxicity assays.

Primary screening was conducted with the MCF-7 D538G and MCF-7 Y537S cells using a standard protocol (48). Specifically, cells were harvested, counted, and then plated to a final density of 600 cells per well in 60 μL of medium in 384-well microtiter plates (Corning, 3707) using a BioTek MultiFlo dispenser (BioTek.). After incubating the assay plates overnight in DMEM medium (Thermo Fisher Scientific) in 10% FBS (Atlanta Biologicals), the Prestwick Collection (1,280 drugs; Prestwick Chemical) were added to each plate to a final compound concentration 3 μM (1 replicate per compound, 0.6% DMSO) using a BioMek FX liquid handler (Beckman Coulter). In confirmation studies, MCF-7 cells harboring the *ESR1* WT, Y537S, and D638D were tested individually in a multidose format (doses: 3, 1, and 0.37 μM with 3 replicates per compound, per dose per cell line). For both primary screen and confirmatory studies, Cell Titer Glo reagent (Promega) was added to each well (10 μL of a 1:2 dilution in PBS/Triton X-100 [1%] final) and mixed after an incubation period of 96 hours under growth conditions. Plates were incubated for 10 minutes at room temperature, and luminescence was determined for each well using an EnVision multilabel plate reader (Perkin Elmer). The assays for all 3 cell lines typically displayed *Z*’ values greater than 0.6 (22).

### In vitro drug combination experiments with ESR1 WT and mutant cell lines.

Pair-wise drug combinations experiments with OTX015 and abemaciclib were conducted with the MCF-7 cell line (*ESR1* WT) and 2 *ESR1* mutant MCF-7 cell lines, each carrying either the D538G or Y537S mutation. Cells from all 3 cell lines were harvested, counted, and then plated to a final density of 600 cells per well in 50 μL of medium in 384-well microtiter plates (3707, Corning) using a BioTek MultiFlo dispenser (BioTek). After incubating the assay plates overnight in DMEM (Thermo Fisher Scientific) in 10% FBS (Atlanta Biologicals) at 37°C in a 5% CO_2_ atmosphere, these drugs were added to each plate to a final compound concentration that ranged from 2.7 × 10^–6^M to 3.99 × 10^–9^M (3 replicates per combination per dose per cell line, 0.3% DMSO final) by Labcyte Echo 655 acoustic dispenser (Beckman) utilizing a dose-response matrix design combination program where all the possible dose combinations for a drug pair can be tested. Sham controls (e.g., OTX015 versus OTX-15 and abemaciclib versus abemaciclib) were included for each cell line, which OTX015 should be additive in the absence of any systematic plate errors. Cell Titer Glo reagent (Promega) was added to each well (10 μL of a 1:2 dilution in PBS/Triton X-100 [1%] final) and mixed after an incubation period of 96 hours under growth conditions. Plates were incubated for 10 minutes at room temperature, and luminescence was determined for each well using an EnVision multilabel plate reader (Perkin-Elmer).

### Analysis of high-throughput screening data and drug-combination studies.

The Genedata Screener software (version 16, Genedata) was used to process and analyze the screening data for small molecules. For analysis of the data from the primary screen of the UT Southwestern chemical library, experimental results obtained from EnVision multilabel plate reader were processed using the Assay Analyzer module of the Genedata Screener Suite. For each plate, the raw data values for all wells were normalized using the following equation, which assumes hits are infrequent, structurally unrelated, and randomly distributed on individual library plates:

Normalized values = (raw values – median of test population)/(median of test population) × 100 (48). 

This assumption proved true for all the library plates. The test population consists of the UT Southwestern library located in columns 3–22 of the 384-well assay plate. A positive control (3 μM OTX final concentration in column 1) was included on every assay plate, as was a neutral control (DMSO only, columns 2 and 23), and untreated cells were at column 24. Normalized well values were then corrected, where a correction factor for each well was calculated using a proprietary pattern detection algorithm in the Assay Analyzer software. *Z* scores were calculated from the corrected normalized activity for each compound (23).

For confirmation studies, the top 30–40 hits from the primary screen were selected, and each compound was assayed in triplicate at the doses described above; the data for each assay well was normalized to the neutral controls. For each compound, the normalized activity values were condensed to a single value (condensed activity) using the “Robust Condensing” method in Genedata Screener. The condensed activity is the most representative single value of the triplicates. In general, the triplicates were precondensed into a pair of values as follows: Values(X,Y) = (Median of Triplicates [m]) ± Dispersion, where Dispersion = Median (|X1 – m|,|X2 – m|,|X3 – m|). The less X and Y differ (|X – Y|), the better the data quality. For data points where |X – Y| *≤* 30%, the median of X and Y was used as the condensed activity, which is also the median of the triplicate measurements. Otherwise, a condensing function Min(X,Y) was used to estimate the condensed activity. Curve fitting for IC_50_ was performed using Smart Fit method in Analyzer module of Genedata Screener (version 15) software suite as previously described (49).

Pairwise drug combinations experiments were analyzed as described previously (50). Briefly, data files from the EnVision Multi-label Plate Reader (Perkin-Elmer) were loaded into the Genedata Analyzer software (version 16.0, Genedata) and normalized as described above. Combinations data were analyzed with the Compound Synergy Extension module, and synergy scores and combination indices were computed using the methods of refs. 51 and 52. In both cases, the Loewe additivity model of synergy was employed.

### PK studies.

Mice were dosed with 50 mg/kg OTX015 formulated in 10% ethanol, 0.1% Tween 80 (MilliporeSigma), and 0.5% methylcellulose (MilliporeSigma). Blood was collected from the submandibular vein and placed into a K2EDTA BD microtainer at 30, 60, and 240 minutes after dosing. Plasma was processed from the collected whole blood by centrifugation for 10 minutes at 9,600*g* at room temperature. An equal volume of methanol containing 150 ng/mL tolbutamide internal standard was added to 0.1 mL of plasma to precipitate plasma protein and to release bound drug. The supernatant was then analyzed by liquid chromatography–tandem mass spectrometry (LC-MS/MS) using an AB Sciex 4000QTRAP mass spectrometer coupled to a Shimadzu Prominence LC. The compounds were detected with the mass spectrometer in multiple-reaction monitoring (MRM) mode by following the precursor-to-fragment ion transitions optimized for the instrument: OTX015 492.1 to 383.1 and tolbutamide 271.2 to 91.2. An Agilent XDB C18 column (50 × 4.6 mm, 5 μm packing) was used for chromatography with the following conditions: Buffer A, dH_2_0 + 5 mM ammonium acetate; buffer B, methanol; 0–0.25 minimum gradient to 2% B, 0.25–2.0 minimum gradient to 98% B, 2.0–3.0 minimum gradient to 98% B, 3.0–3.5 minimum gradient to 2% B; 3.5–4.0 minimum gradient to 2% B. A value of 3-fold above the signal obtained in the blank plasma was designated the limit of detection (LOD). The limit of quantitation (LOQ), defined as the lowest concentration at which back calculation yielded a concentration within 20% of the theoretical value and above the LOD signal, was 0.5 ng/mL. PK parameters were determined using the noncompartmental analysis tool in Phoenix WinNonlin (Certara Corp.). Sparse sampling was applied, and a user-defined time range of 60–240 minute was used to determine λZ for calculation of half-life. A linear trapezoidal linear interpolation was used for calculation of AUC. T_max_ and C_max_ were determined by visual inspection.

### Xenograft studies.

For β-estradiol–independent xenograft growth assays, MCF-7 cells harboring the WT, Y537S, and D538G mutations and stably transfected with a luciferase reporter were suspended in 1:1 (volume) matrigel/DMEM (Thermo Fisher Scientific) to a final concentration of 5 × 10^7^ cells/mL. In total, 100 μL cell-gel mixture was then injected s.c. into the flank of 6- to 8-week-old ovariectomized, female NOD-SCID mice (UT Southwestern Breeding Core). No exogenous β-estradiol supplementation was provided to mice. Xenograft growth was monitored by luciferase activity using IVIS Lumina (PerkinElmer) following ocular injection of 100 μL of 5 mg/mL potassium luciferin (Gold Bio, LUCK-1G). The experiment was terminated at 6 weeks, and final volumes of isolated tumors were calculated using the formula: volume = (π/6 × larger diameter × [smaller diameter]^2^). For β-estradiol–independent xenograft growth assays using T-47D cells, a similar protocol was followed using ovariectomized female athymic nude mice. Following caliper measurements, tumor volumes were calculated as described above.

For therapeutic studies, 10 million MCF-7 Y537S cells or Y537S PDX (WHIM20) cell suspension in 1:1 (volume) matrigel/DMEM was injected s.c. into the flank of 6- to 8-week-old ovariectomized, female nude mice (Charles River Laboratories) for cell line xenografts or NOD-SCID mice (UT Southwestern breeding core) for PDX. No exogenous β-estradiol supplementation was provided to mice. When tumors reached a size of 100–200 mm^3^, the mice were randomized to various treatment arms. For synergy studies, tumors were allowed to reach a size of 300–400 mm^3^ before they were randomized to one of the indicated treatment arms. The drug dosing schedule was as follows: vehicle oral gavage daily, 6 days/week; OTX015 (100 mg/kg) by oral gavage daily, 6 days/week; abemaciclib (50mg/kg) by oral gavage daily, 6 days/week; and fulvestrant 1 mg by s.c. injection weekly.

### PDE studies.

PDX model WHIM20 was established by direct engraftment of tumor fragments implanted via intramammary fat pad in SCID mice. Tumors were excised at 1,000 mm^3^ and dissected into 2 mm cubes to be cultured ex vivo. Explant was incubated on gelatin sponges for 48 hours in culture medium containing 10% FBS and 1% penicillin/streptomycin with either vehicle or 10 μM of OTX015, JQ1, or I-BET762. Explant tissue samples were fixed in 10% formalin at room temperature overnight and subsequently processed into paraffin blocks. For IHC studies, tissue sections were blocked in Background Sniper (Biocare Medical) followed by overnight incubation at 4°C with Ki-67 (1:500) primary antibody, followed by secondary antibody (1:1,000) incubation for 60 minutes at room temperature. Immunoreactivity was visualized by using DAB (Vector Labs). A proliferative index was calculated as the percentage of Ki-67^+^ cells in 3 randomly selected microscopic fields, magnified at 40× per slide.

### Quantitative PCR (qPCR).

The frozen PDX tumors were ground in RNA extraction buffer, and total RNA was isolated using RNA extraction Kit (MilliporeSigma). For gene transcription analysis, cDNA synthesis was performed with 0.5–1 μg total RNA of WHIM20 tumors using SuperScript III First Strand Synthesis System (Thermo Fisher Scientific). qPCR was perform in Bio-Rad CFX900 using SsoAdvanced Universal SYBR Green Supermix (Bio-Rad). Primers of human GREB1 and GAPDH were purchased from MilliporeSigma.

Sequences of primers were as follows: GREB1: forward, 5′-GTGGTAGCCGAGTGGACAAT-3′; reverse, 5′-ATTTGTTTCCAGCCCTCCTT-3′; and GAPDAH: forward, 5′-GTCTCCTCTGACTTCAACAGCG-3′; reverse, 5′-ACCACCCTGTTGCTGTAGCCAA-3′.

### Statistics.

Each experiment was performed with biological replicates as indicated. Statistical analyses were performed using GraphPad Prism 7.0 (GraphPad Software). A 2-tailed Student’s *t* test was used for comparison between experimental groups. For experiments that involved more than 2 groups, to adjust for multiple comparions, 1-way ANOVA with Dunnett’s test was used to compare various experimental arms with the control arm. When comparing various treatment groups with each other, nonparametric Wilcoxon rank-sum test with Bonferroni correction was used. *P* < 0.05 was considered statistically significant.

### Study approval.

All animal experiments were performed in accordance with UT Southwestern IACUC approved protocol.

## Author contributions

PGA and GPG conceptualized the study. SNU, QW, SK, SG, NSW, GPG, SW, BAP, and PGA designed the study methodology. SNU, QW, SK, SYW, SG, MH, KP, and SW conducted experiments. SNU, QW, SK, VSM, SW, BAP, SG, NSW, YL, GVR, GPG, and PGA interpreted data and/or performed statistical analysis. SNU, QW, SK, VSM, SG, NSW, YL, JKS, BAP, RSM, SM, JML, GVR, RJ, MSW, BHP, GPG, AMC, CMC, and PGA contributed to the writing, review, and/or revision of the manuscript. PGA supervised the study.

## Supplementary Material

Supplemental data

Supplemental table 1

Supplemental table 2

Supplemental table 3

Supplemental table 4

## Version 1 07/26/2022

In-Press Preview

## Version 2 09/08/2022

Electronic publication
