# Supplementary material for: Targeting ESR1 mutation–induced transcriptional addiction in breast cancer with BET inhibition
Source: JCI Insight. 2022 Sep 8;7(17):e151851. doi: 10.1172/jci.insight.151851 (PMC9536271; doi:10.1172/jci.insight.151851)
Supplement: Supplemental data [file jciinsight-7-151851-s147.pdf]

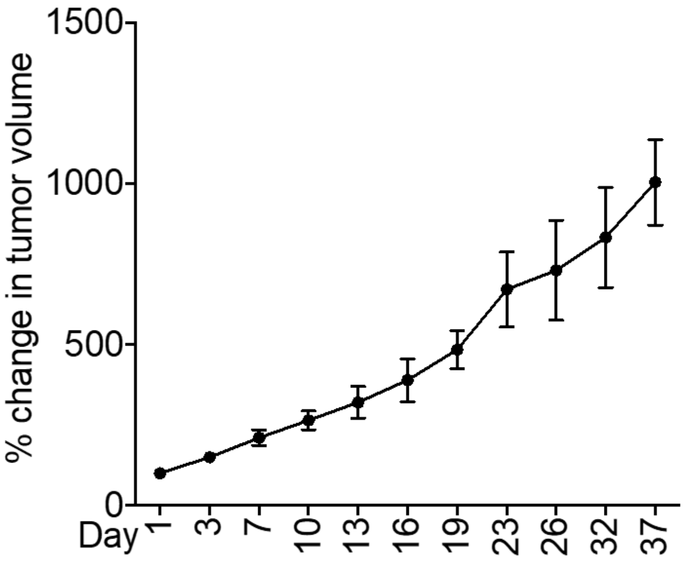

Figure S1A. β-estradiol supplementation rescues growth of xenografts derived from MCF-7 cells harboring wild-type *ESR1* in ovariectomized mice: MCF-7 cells harboring wild-type *ESR1* were injected (5 million cells/injection) subcutaneously into the flanks of ovariectomized, 6-week old, female athymic, nude mice (n= 6 tumors) after subcutaneous implantation of 0.17 mg, 2-week release β-estradiol pellet at a site distant from the tumor injection site. Tumors were allowed to grow and tumor volumes were quantified twice a week. The results were plotted as average tumor volume ± SEM.

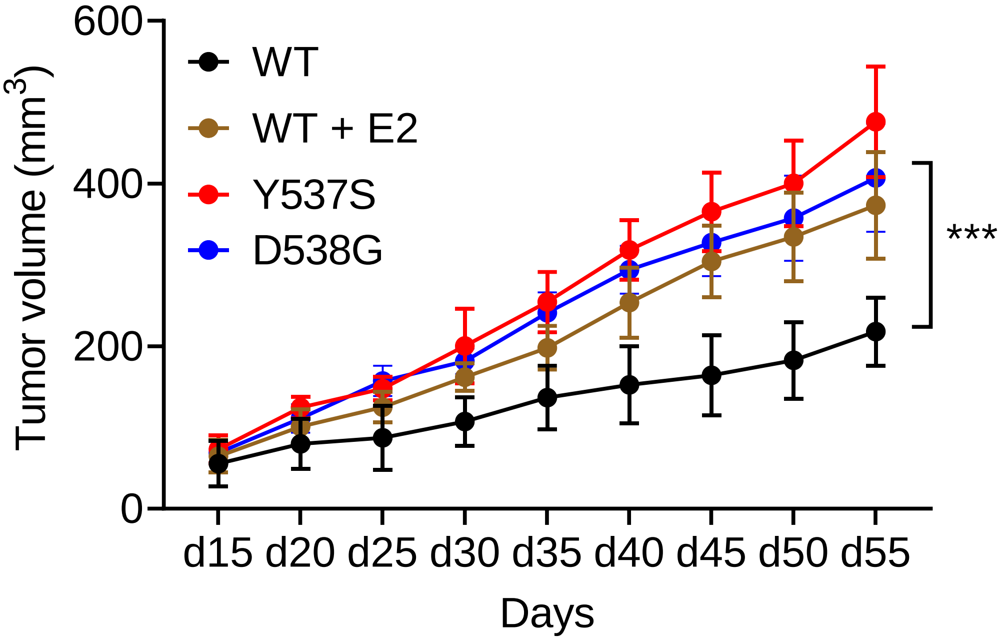

Figure S1B. *ESR1* mutations confer growth advantage in xenografts derived from T-47D cells when grown in ovariectomized, female athymic nude mice without exogenous estradiol supplementation: T-47D cells harboring *ESR1* Y537S or D538G mutations were injected (5 million cells/injection) subcutaneously into the flanks of ovariectomized, 6-week old, female athymic, nude mice (n= 10 tumors). T-47D cells harboring wild-type *ESR1* were similarly injected (5 million cells/injection) into mice with or without 0.17 mg, 2-week release β-estradiol. Tumors were allowed to grow and tumor volumes were quantified as shown. The results were plotted as average tumor volume ± SEM. ANOVA with Dunnett's test was used to make multiple comparisons using WT tumors as control. *** ≤ 0.0005.

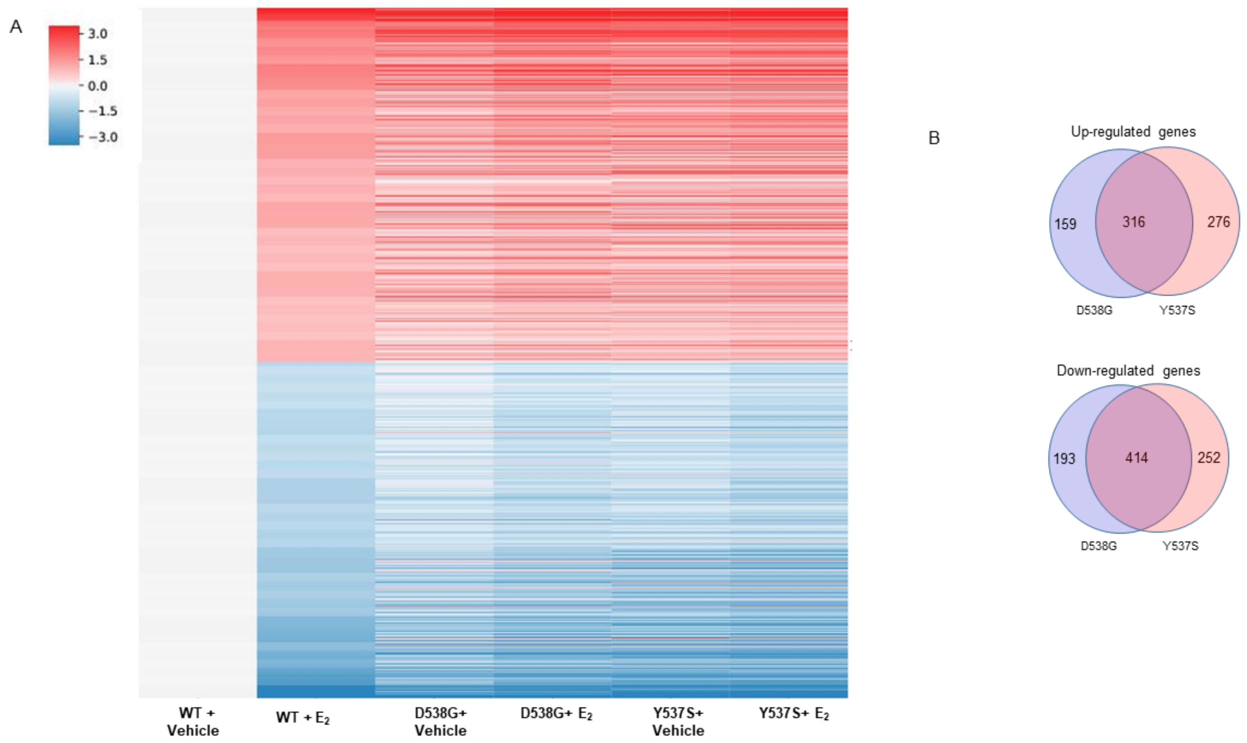

Figure S2. Ligand-independent activation of ERα signaling in *ESR1* mutant MCF-7 cells: (A) Heat map showing normalized $\log_2$ fold change in expression of genes in the wild-type cells that have been hormone-depleted for 3 days and treated with vehicle or 10 pM β-estradiol (to define estrogen-responsive genes). Also shown is $\log_2$ fold change in expression of the same genes in MCF-7 *ESR1* D538G and Y537S cells treated with vehicle or 10 pM β-estradiol after hormone deprivation. (B) Venn diagrams depicting overlap between upregulated/downregulated genes in MCF-7 Y537S and D538G cells. E_2= β-estradiol.

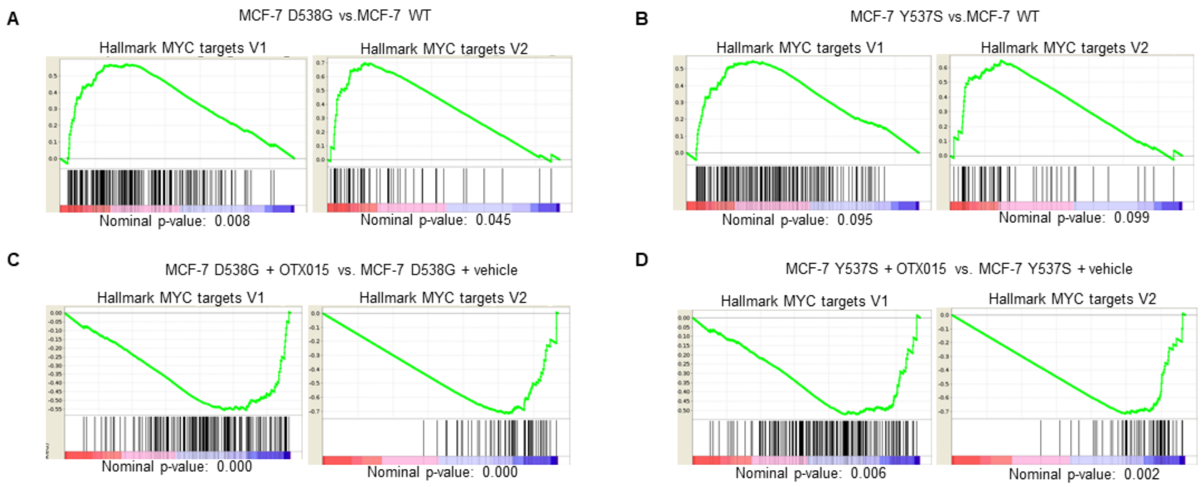

Figure S3. OTX015 reversed the transcriptional programs stimulated by the Y537S and D538G mutations: (A-B) GSEA of genes differentially expressed in MCF-7 Y537S and D538G cells relative to the wild-type cells showing activation of MYC signaling. (C-D) GSEA of genes differentially expressed in MCF-7 Y537S and D538G cells following treatment with OTX015 showing suppression of MYC signaling.

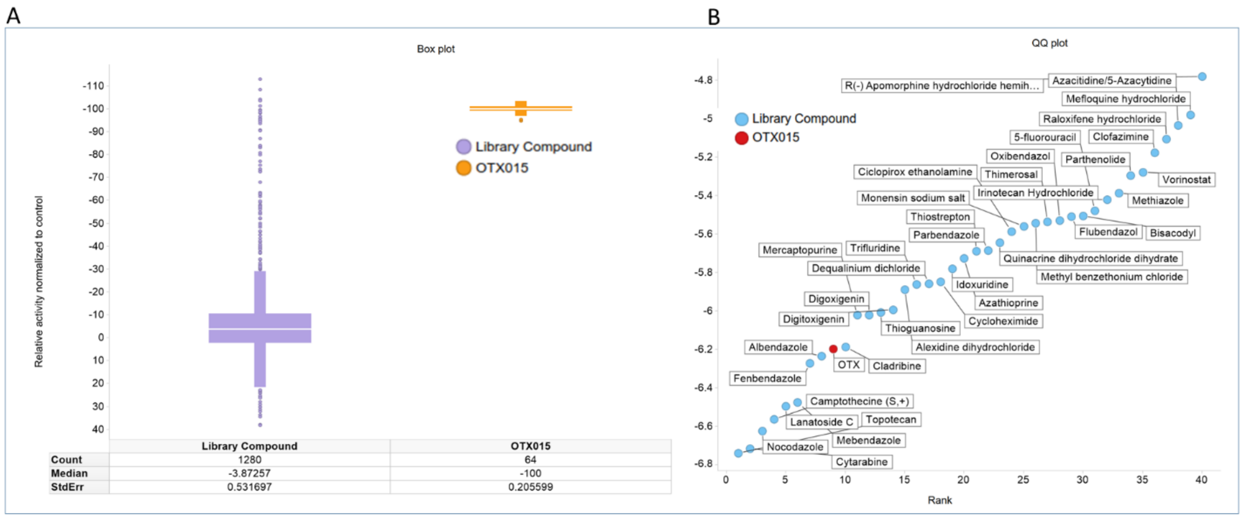

Figure S4. OTX015 is a top inhibitor of *ESR1* mutant cells relative to drugs in the Prestwick chemical library: (A) A plot of relative activity of individual compounds in the Prestwick chemical library relative to OTX015 performed MCF-7 D538G cells. The screen was carried out at a drug concentration of 3 μM in 384-well format. Cytotoxicity was assayed by luminescence using Cell Titer Glo^{TM}. (B) A plot of log IC_{50} values of the top 40 hits identified in the primary screen.

Parameter	Unit	Estimate
$T_{1/2}$	Hr	4.1
T_{max}	Hr	0.5
C_{max}	mg/L	1706
AUC	mg*hr/L	8678

Figure S5. Pharmacokinetic parameters of OTX015: Plasma pharmacokinetics of OTX015 administered PO at 50 mg/kg. Pharmacokinetic parameters were determined using the non-compartmental analysis tool in Phoenix WinNonlin (Certara Corp., Princeton, NJ.) Sparse sampling was applied and a user defined time range from 60-240 min was used to determine Lambda Z for calculation of half-life. A linear trapezoidal linear interpolation was used for calculation of area-under-the-curve (AUC).

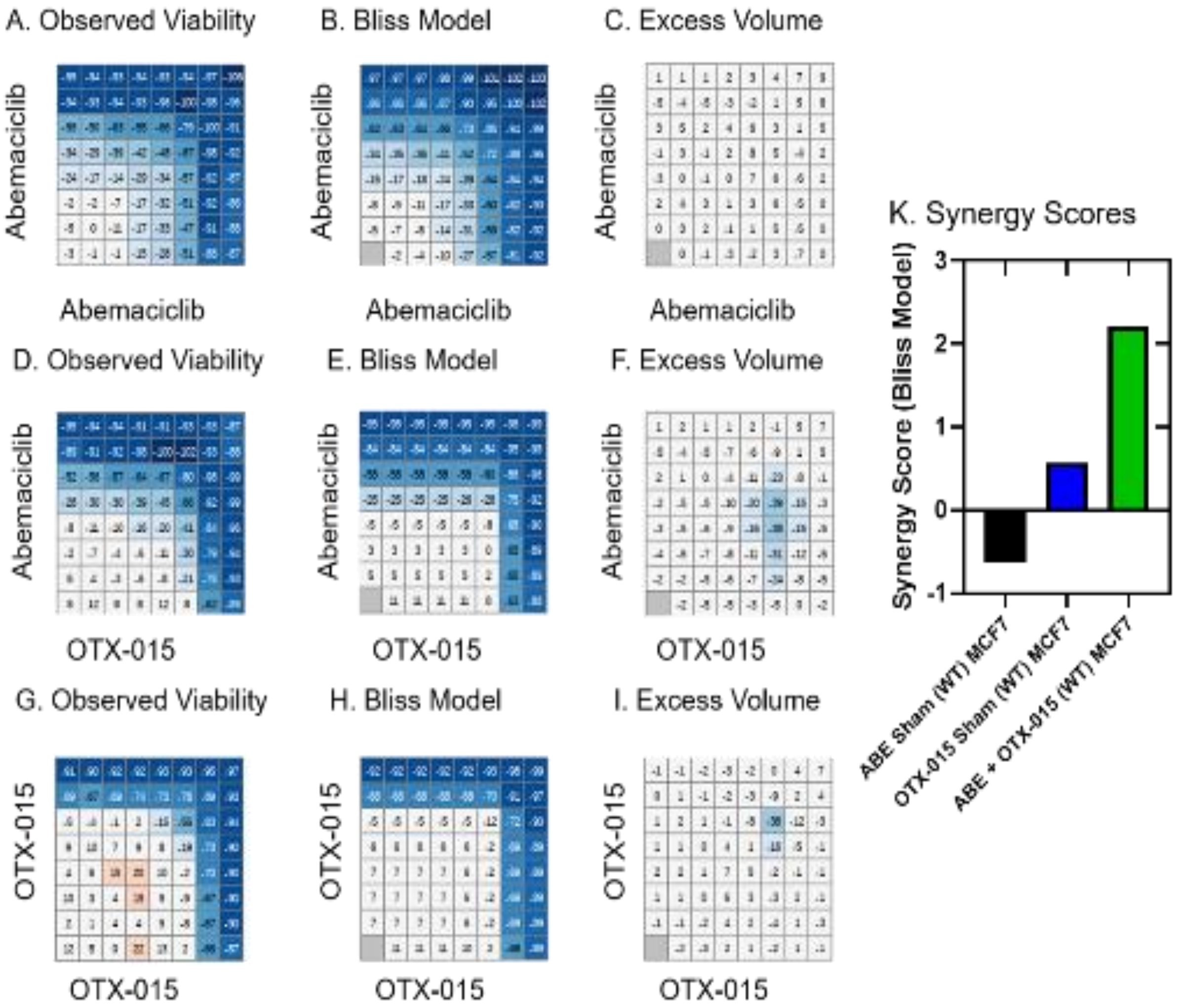

Figure S6. BET inhibition synergizes with abemaciclib in inhibiting the growth of MCF-7 cells harboring wild-type *ESR1*: Combination matrices were prepared in 384 well microtitier plates and WT MCF7 cell viability was tested under the following conditions: (A) abemaciclib vs. abemaciclib (sham), (D) abemaciclib vs. OTX15, and (G) OTX015 vs. OTX015 (sham). Concentrations of each drug increase from left to right (0.004, 0.011, 0.033, 0.1, 0.3, 0.9, and 2.7 µM) and from bottom to top (0.004, 0.011, 0.033, 0.1, 0.3, 0.9, and 2.7 µM) with zero drug in the lower left-hand corner of each matrix. The additive condition was calculated for each experiment using the Bliss model (B, E, and H). Excess volume (Observed – Model) was calculated for each experiment (C, F, and I) and synergy scores were determined (J).

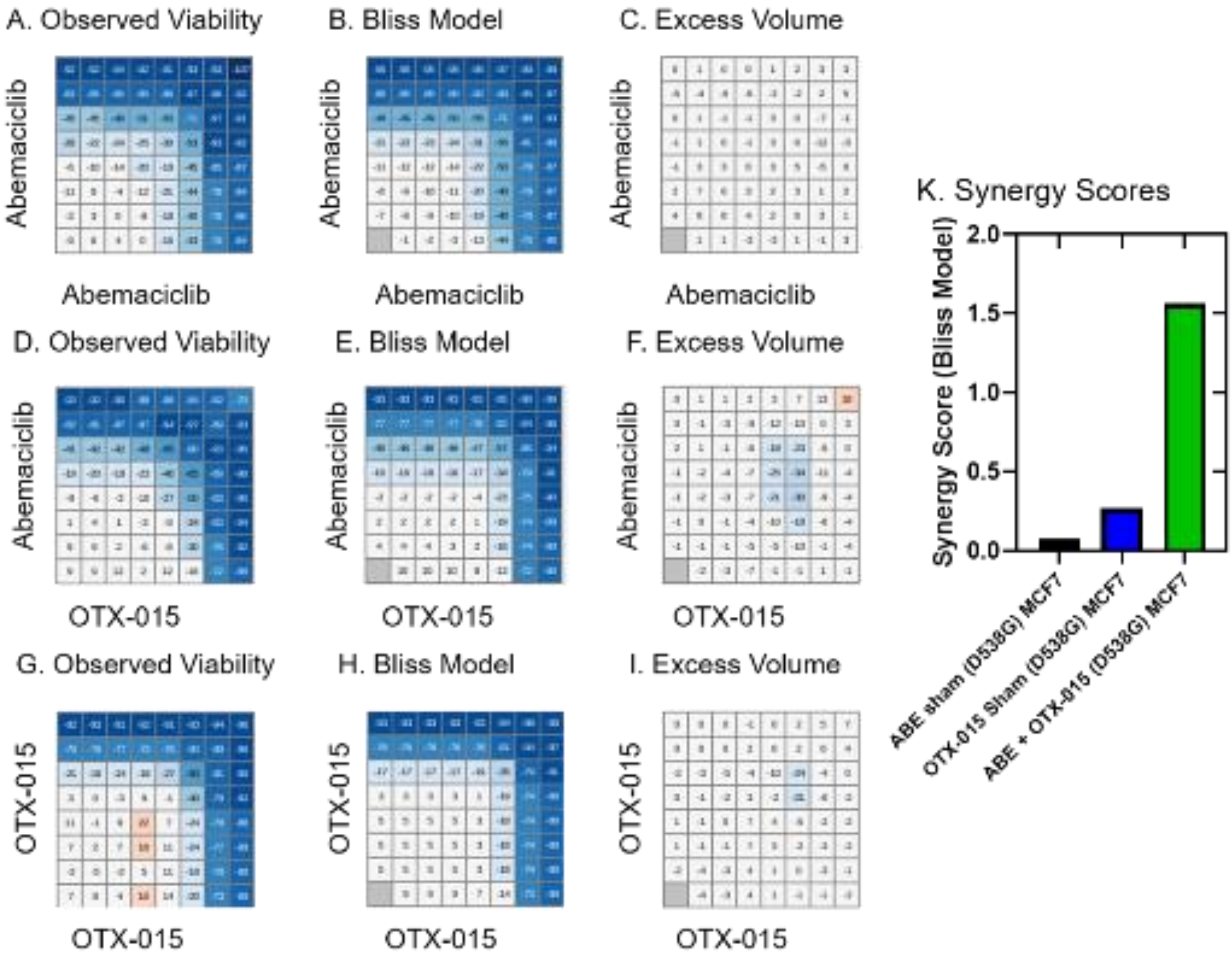

Figure S7. BET inhibition synergizes with abemaciclib in inhibiting the growth of MCF-7 cells harboring the D538G mutation: Combination matrices were prepared in 384 well microtitier plates and MCF7 D538G cell viability was tested under the following conditions: (A) abemaciclib vs. abemaciclib (sham), (D) abemaciclib vs. OTX15, and (G) OTX015 vs. OTX015 (sham). Concentrations of each drug increase from left to right (0.004, 0.011, 0.033, 0.1, 0.3, 0.9, and 2.7 µM) and from bottom to top (0.004, 0.011, 0.033, 0.1, 0.3, 0.9, and 2.7 µM) with zero drug in the lower left-hand corner of each matrix. The additive condition was calculated for each experiment using the Bliss model (B, E, and H). Excess volume (Observed – Model) was calculated for each experiment (C, F, and I) and synergy scores were determined (J).
